# A large-scale stochastic spatiotemporal model for *Aedes albopictus*-borne chikungunya epidemiology

**DOI:** 10.1371/journal.pone.0174293

**Published:** 2017-03-31

**Authors:** Kamil Erguler, Nastassya L. Chandra, Yiannis Proestos, Jos Lelieveld, George K. Christophides, Paul E. Parham

**Affiliations:** 1 Energy, Environment and Water Research Center, The Cyprus Institute, 2121 Aglantzia, Nicosia, Cyprus; 2 Department of Infectious Disease Epidemiology, Faculty of Medicine, Imperial College London, London W2 1PG, United Kingdom; 3 Department of Atmospheric Chemistry, Max Planck Institute for Chemistry, D-55128 Mainz, Germany; 4 Department of Life Sciences, Faculty of Natural Sciences, Imperial College London, South Kensington Campus, London SW7 2AZ, United Kingdom; 5 Computation-based Science and Technology Research Center, The Cyprus Institute, 2121 Aglantzia, Nicosia, Cyprus; 6 Department of Public Health and Policy, Faculty of Health and Life Sciences, University of Liverpool, Liverpool L69 3GL, United Kingdom; Universita degli Studi di Camerino, ITALY

## Abstract

Chikungunya is a viral disease transmitted to humans primarily via the bites of infected *Aedes* mosquitoes. The virus caused a major epidemic in the Indian Ocean in 2004, affecting millions of inhabitants, while cases have also been observed in Europe since 2007. We developed a stochastic spatiotemporal model of *Aedes albopictus*-borne chikungunya transmission based on our recently developed environmentally-driven vector population dynamics model. We designed an integrated modelling framework incorporating large-scale gridded climate datasets to investigate disease outbreaks on Reunion Island and in Italy. We performed Bayesian parameter inference on the surveillance data, and investigated the validity and applicability of the underlying biological assumptions. The model successfully represents the outbreak and measures of containment in Italy, suggesting wider applicability in Europe. In its current configuration, the model implies two different viral strains, thus two different outbreaks, for the two-stage Reunion Island epidemic. Characterisation of the posterior distributions indicates a possible relationship between the second larger outbreak on Reunion Island and the Italian outbreak. The model suggests that vector control measures, with different modes of operation, are most effective when applied in combination: adult vector intervention has a high impact but is short-lived, larval intervention has a low impact but is long-lasting, and quarantining infected territories, if applied strictly, is effective in preventing large epidemics. We present a novel approach in analysing chikungunya outbreaks globally using a single environmentally-driven mathematical model. Our study represents a significant step towards developing a globally applicable *Ae. albopictus*-borne chikungunya transmission model, and introduces a guideline for extending such models to other vector-borne diseases.

## Introduction

Chikungunya is an incapacitating disease caused by the recently re-emerging Chikungunya virus (CHIKV) [1], which is transmitted to humans via mosquitoes of the *Aedes* genus. Since its first identification in Tanzania in 1952, CHIKV has been detected in both low- and high-income countries, causing social and economic burdens. As the disease appears not to be directly associated with poverty or wealth, the recent rapid spread is thought to be the result of increased global travel, trading across international borders, and climate change, allowing known vectors to be transported quickly to new habitats [2]. Over the last 10 years, the geographic range of CHIKV has expanded [3] and in early 2005, outbreaks occurred in Mayotte, Mauritius, and the Comoro islands. By late April, Reunion Island had also been affected and a year later, approximately 244,000 cases and 203 reported deaths had occurred [4]. Epidemics regularly occur in India, with an estimated one million cases reported during the 2006 epidemic, while repeated smaller-scale epidemics have occurred since 2005 in many other Asian and African countries [5].

The first reported outbreak of CHIKV in continental Europe was in Italy in 2007 [6] in the adjacent villages of Castiglione di Cervia and Castiglione di Ravenna in North East Italy. It is believed that the virus was introduced by a man travelling from India to visit family members [7]. The outbreak was well-contained overall, with 205 confirmed cases and only one reported death [7–9]. CHIKV was identified in South East France in September 2010 [10], and imported cases have also been detected in British Guyana, French Guiana and other countries of the West Indies, Germany, Norway, China, and the Philippines [11]. The recent establishment of *Ae. albopictus* in Spain poses a great threat for CHIKV emergence in Spain [12].

The first local transmission of CHIKV in United States was detected in Caribbean territories in 2013 [13] although *Aedes aegypti* was identified as the main vector in these outbreaks [14]. Since 2014, local transmission have also been detected in Florida, Puerto Rico, and the U.S. Virgin Islands [13]. *Ae. aegypti* is also responsible for the recent explosive outbreaks of the Zika virus in Latin America [15] and its introduction to U.S. in 2016 [16].

Once a CHIKV-infected vector bites a human host and transmits the virus, an asymptomatic incubation period begins, ranging from one to twelve days with an average of two to four days [1, 11]. Following this incubation period, 15% of infections remain asymptomatic [1, 3], while others experience symptoms corresponding to the acute stage of infection, often including fever (temperatures above 39°C), arthralgia in small and large joints, and body rash, while vomiting and nausea are also common [3, 11, 17]. Acute symptoms generally last seven to ten days, but associated aches and pains can be long-lasting in 30% of patients marking the chronic stage of the infection [17]. Continuing long-term inflammation of the joints and connective tissue during the chronic stage may have disabling consequences similar to rheumatoid arthritis [3, 11]. Chikungunya is rarely fatal, but deaths can occur in the young, elderly, or those immunosuppressed or immunocompromised [3, 11, 17].

The 2005-2006 and 2007 chikungunya outbreaks in the Indian Ocean and Italy, respectively, are thought to have been associated with a recent mutation of alanine-226 in the CHIKV envelope protein to valine, which increased the infectiousness of CHIKV in *Ae. albopictus* compared to *Ae. aegypti* due to more efficient attachment and migration from the midgut to the salivary glands [3, 18].

*Ae. albopictus* can successfully inhabit both tropical and temperate climates [19–21], with its wide geographic distribution arising from its ability to inhabit both rural and urban settings and display both anthropophilic and zoophilic behaviour [22]. It is known as a container breeder due to the variety of natural and artificial environments used as breeding sites [23, 24]. Adults are generally active during the day, but can also feed at night; in addition, although they prefer to feed outdoors, they do bite indoors, making prevention more problematic. Trading of car tires and timber from Asia was the probable route of introduction into countries where its presence would otherwise have been unlikely [3]; eggs are resistant to desiccation and are thus likely to survive the travelling time. In addition, the temperate strain of *Ae. albopictus*, in contrast to the tropical strain, can lay diapausing eggs with elevated cold-resistance contributing further to the reproductive success in colder climate zones [25–27].

The global distribution and population dynamics of *Ae. albopictus* are highly influenced by environmental variables [28]; temperature significantly affects the mosquito developmental and metabolic rates, while it is thought to also influence egg production and the frequency of blood meals, with extreme temperatures (>40°C) having damaging effects. Rainfall is tightly associated to breeding sites of aquatic immature mosquito stages, with heavy rainfall compromising breeding sites or sweep away the immature stages. Reviews of how climatic variables affect *Ae. albopictus* biology have been previously published [22, 29, 30] and the roles of climate (and climate change) on vector-borne diseases have been reviewed [31].

Mathematical models of infectious diseases enable predictions of and insights into epidemic dynamics to help guide public health planning and response [32–34]; however, relatively few validated CHIKV transmission models have been developed to date and even fewer based on validated *Ae. albopictus* population dynamics models [22, 35], making reliable predictions about transmission and the potential impacts of different intervention programs at local and global scales challenging. We have previously developed an environmentally-driven model of *Ae. albopictus* population dynamics and used a Bayesian approach to assess model fit to vector surveillance data from Emilia-Romagna, Italy [30]. Our model has demonstrated encouraging predictive capacity over the region and beyond. In the current study, we aimed to use this model as the basis for developing a stochastic spatiotemporal model of *Ae. albopictus*-driven CHIKV transmission using large-scale gridded environmental datasets. Next, we sought to use this integrated model to investigate the CHIKV outbreaks in Italy [7–9] and on Reunion Island [4, 36], as well as to infer more general and valuable insights into CHIKV transmission dynamics and the impact of vector and outbreak management strategies.

## Methods

**Fig 1:**
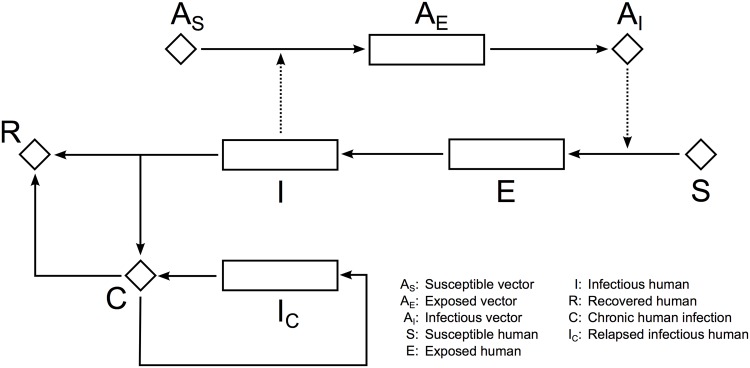
Flow diagram of the stochastic *Ae. albopictus*-borne CHIKV transmission model. Rectangles indicate states with durations, such as incubation periods, while diamonds indicate states with daily survival but without development/incubation periods. Solid arrows indicate state transformation, while dashed arrows indicate modulation/influence.

We developed a discrete-time stochastic susceptible / exposed / infected / recovered / chronic-stage (SEIRC) model for *Ae. albopictus*-borne chikungunya transmission. A schematic representation of the model and an abbreviated list of parameters are given in Fig 1. An expanded parameter list is given in Table 1 together with domain boundaries (and references, where applicable) for parameter inference.

**Table 1:** Model parameters with reference intervals obtained from literature.

Parameter	Definition	Prior interval	References
*ω*_*ν*_	Average latent period in vectors	0-10 days	[38–40]
*σ*_*ω*_*ν*__	Standard deviation of *ω*_*ν*_	0-5 days	
*ω*	Average latent period in humans	0-10 days	[36, 38, 40–42]
*σ*_*ω*_	Standard deviation of *ω*	0-5 days	
*γ*	Average infectious period in humans	0-10 days	[38, 40, 41, 43]
*σ*_*γ*_	Standard deviation of *γ*	0-5 days	
*α*_scpH_	Human susceptibility to infections	0-1	[38, 40]
*α*_scpV_	Vector susceptibility to infections	0-1	[40, 44]
*p*_*R*_	Probability of recovery following the initial infectious period	0-1	[45]
*p*_*C*_	Probability of a persistent infection following the initial infectious period (1-*p*_*R*_)	0-1	[45]
*p*_*CR*_	Daily probability of recovery from a chronic infection	0-1	
*p*_*CI*_	Daily probability of relapse during a chronic infection (*p*_*CR*_+*p*_*CI*_ ≤ 1)	0-1	
*α*_*η*_	Fraction of symptomatic infections which are also reported	0-1	[40, 46–49]
*α*_ovt_	Average number of ovitraps per km^2^	0-10^3^	[50]
*k*_hum_	Average number of bites per human	0-100	
*k*_vec_	Average number of bites per vector and daily fecundity	0-1	
*q*_*i*_	Probability of a person to reside in patch *i* for a day	0-1	
*c*	Degree of separation among patches	0-5	
*t*_intro_	Time of introduction of an infectious person (with regards to the beginning of simulation)	1-365 days	
*h*	The patch where an infectious person is introduced	indices of available patches	

We implemented the model in ANSI C and developed a Python interface to run and facilitate integration with various environmental data sets. The code and related material are available in the albopictus Python package (v.0.8), which is available from the Python package index [37].

### Environmental data sets

We adopted a large-scale modelling approach by focusing on the cells of gridded environmental datasets. We used the gridded climate dataset (E-OBS) of the EU-FP6 project ENSEMBLES with 0.25° resolution [51] (about 25 km along the equator) and the UN-adjusted Gridded Population of the World Version 3 (GPWv3) with matching resolution for 2005 [52] as the source of temperature, precipitation and human population density for simulations over Europe. Throughout the text, we refer to this environmental dataset as ClimEurope.

In addition, we employed the ECHAM5/MESSy2 Atmospheric Chemistry (EMAC) general circulation model [20, 53, 54] to establish global datasets available over Reunion Island. Climatic boundary conditions using AMIP-II [55] sea-surface temperature (sst) and sea-ice coverage (sic) assimilation data was imposed to this model, which has a resolution of 0.5° latitude and longitude (about 50 km grid spacing along the equator). The model was evaluated against meteorological re-analysis data and was also used for future climate projections [20]. We refer to this dataset as ClimGlobe throughout the text.

### Vector dynamics model

We represented vector dynamics with the recently developed environmentally-driven population dynamics model of *Ae. albopictus*, as described in Erguler *et al.* 2016 [30]. Throughout the text we refer to this as the albopictus model.

As an improvement to the original model, we implemented a more realistic age-dependent survival process for adult stages. We described adult survival time with a gamma distribution [56], and calculated daily survival, *p*_4_, of a *d*-day old vector with

$$\begin{array}{c} p_{4}=1-\frac{F(d+1;\mu ,\sigma )-F(d;\mu ,\sigma )}{1-F(d;\mu ,\sigma )}, \end{array}$$ (1)

where *F*(⋅) is the cumulative distribution function of the gamma distribution with shape and scale parameters adjusted to set the mean and standard deviation of the distribution *μ* and *σ* days, respectively (see S1 Text for a detailed derivation).

We followed the assumption of temperature-dependent adult survival in the original model and performed parameter inference over the Emilia-Romagna surveillance dataset [50]. We present the emerging optimum posterior mode [30] in S2 Text. The parameters sampled from this posterior mode, Θ4, are labelled as “Q4” in the albopictus Python package (v.0.8) [37].

Overall, we observed that the model with Θ4 suggests elevated cold resistance for diapausing eggs and the involvement of both temperature and photoperiod in the process of diapause (see S2 Text for a detailed account of the results). The threshold for cold resistance in adult vectors is around -2°C, while diapausing eggs can be expected to have 50% daily survival probability at -10°C or lower. While the data are not restrictive on the lower bound of cold resistance, they necessitate that diapausing eggs should resist very low temperatures. We note, however, that these temperatures are daily averages and they represent an entire grid cell of the environmental datasets. According to Θ4, diapause entry is triggered by a drop in temperature (below 11.59 ± 0.003°C), while exit from diapause is mainly initiated by the duration of daylight exceeding 11.78 ± 0.001 hours. We found that, with regards to its biological implications, Θ4 closely resembles the posterior mode Θ1 in Erguler *et al.* 2016 [30].

### Local transmission model

Chikungunya virus is transmitted from one person to another via the bites of infectious vectors [11]. Here, we modelled the initiation of disease transmission with a susceptible vector becoming infected upon receiving a blood meal from an infectious human. When infected, we assumed that the vector passes to the exposed stage, *A*_*E*_, where it spends an incubation period of approximately *ω*_*ν*_ days. Following the incubation period, the vector becomes infectious, *A*_*I*_, and spends the rest of its life in this stage [38–40].

We assumed that an infected human passes to the exposed stage, *E*, and, after approximately *ω* days, becomes infectious, *I* [36, 38, 40–42]. We followed previous modelling approaches [40, 46–49] by assuming that an infection is captured by a surveillance system with a certain probability, *α*_*η*_, whereby a person becomes infectious and thus symptomatic. The infectious stage lasts approximately *γ* days, and is either followed by recovery, *R* [38, 40, 41, 43], or progression to the chronic stage, *C* [57–59]. We defined fixed daily probabilities, *p*_*R*_ and *p*_*C*_, respectively, for each of these routes.

Upon recovery or entrance to the chronic stage, we assumed that a patient is no longer infectious; however, in the chronic stage, which could last for several years, spontaneous remission of the infectious stage might occur [45, 60]. We modelled such sporadic relapses by allowing transformations to an alternative infectious stage, *I*_*C*_. Unlike the initial infectious stage, this alternative stage transforms back to the chronic stage, and the chronic stage transforms to the recovered stage with a small but fixed daily probability, *p*_*CR*_. Due to the lack of detailed accounts of environmental dependence of relapses from the chronic stage, we adopted a fixed daily probability of relapse, *p*_*CI*_.

In order to model latent and infectious periods, we used an age-structured development model as in Erguler *et al.* 2016 [30]; however, instead of assuming a fixed duration, which would be suitable for modelling average development time in a deterministic context, we describe the lengths of latent and infectious periods by a gamma distribution [56]. Similarly to Eq (1), latent and infectious stages progress every day with a probability of completion given by

$$\begin{array}{c} p_{d}=1-\frac{F(d+1;\mu ,\sigma )-F(d;\mu ,\sigma )}{1-F(d;\mu ,\sigma )}, \end{array}$$

where *F*(⋅) is the cumulative distribution function of a *d*-day old infection with mean *μ* and standard deviation *σ*. According to this, the duration of each stage is described by the mean and the standard deviation of the gamma distribution. In this context, development follows survival, that is, survival is independent of development and the development process only applies to vectors surviving up to day *d*.

We assumed that chikungunya infection does not significantly elevate human mortality rate [4, 9], and also, human population size is unchanging during the time-frame of an outbreak. In contrast, we modelled the survival of exposed and infectious vectors with an age- and temperature-dependent gamma distribution within the stochastic context. That is, we estimated daily survival probabilities of adult females with Eq (1) and obtained the daily number of surviving vectors from a binomial distribution with *p*_4_ as the probability of success.

The rate of viral transmission between human and vector populations depends on the number of interactions between the two species. The assumption of rapid mixing, where human and vector populations freely and randomly interact with each other, is implicit in canonical deterministic outbreak models and their stochastic derivatives [38, 40, 49, 61–63]. Although active mobility of *Ae. albopictus* is limited to a short range [64], humans can quickly move over large distances and give rise to rapid mixing [65]. By relying mainly on human mobility, we explicitly assumed rapid mixing and regarded human-vector interactions as a Poisson process where, on average, *λ* interactions—successful blood meals—take place between the two species every day. The average number of interactions is independent of their respective disease states and transmission occurs only whenever either vector or human are infectious.

Although *Ae. albopictus* has a strong affinity for humans, it demonstrates a wide range of host preference [66, 67]. In the absence of humans, it will obtain blood meals from other mammals and non-mammal species including birds and reptiles [68]. In the albopictus model, we assumed that the number of eggs laid by female *Ae. albopictus* (fecundity) is independent of human population density [30]. In accordance with this, here, we assumed that fecundity and the number of human-vector interactions are independent quantities; *Ae. albopictus* will always find the blood meal it needs even if there are no humans around.

A large number of disease transmission models [38, 40, 49, 69–71] have made use of the underlying assumption that the rate of human-vector iteractions can be described by the biting rate of a vector (*b*: the number of bites per vector per unit time) and the total number of vectors (*nv*_total_ = *nv*_*As*_ + *nv*_*Ae*_ + *nv*_*Ai*_). By analogy, we write the daily average number of bites as

$$\begin{array}{c} \lambda_{V}=b\;\times \;nv_{\text{total}}, \end{array}$$

where the time unit of *b* is one day. This equation implies that a vector successfully obtains, on average, *λ*_*V*_ blood meals from humans every day given unlimited supply of the host. The standard model is valid only when the number of humans is not a limiting factor. When humans are outnumbered by large vector populations, or when too few humans are present in vector habitats, mosquitoes are forced to seek alternative sources of blood either due to competition or difficulty in finding a human host. The result is the plateauing of the number of human-vector interactions. In order to account for this saturation effect, we defined *k*_hum_ as the number of bites per human per unit time, where the daily average number of bites a person receives is

$$\begin{array}{c} \lambda_{H}=k_{\text{hum}}n_{\text{total}}, \end{array}$$

where *n*_total_ is the total number of humans (*n*_*S*_ + *n*_*E*_ + *n*_*I*_ + *n*_*Ic*_ + *n*_*R*_). This equation implies that enough mosquitoes are present to deliver the daily average number of bites a person receives; therefore, it applies mostly to the opposite side of the spectrum when *nv*_total_ is much larger than *n*_total_.

We argue that an appropriate model for the number of vector-host interactions would be neither *λ*_*V*_ nor *λ*_*H*_, but the harmonic mean of the two [72, 73],

$$\begin{array}{c} \lambda =\frac{2\lambda_{V}\lambda_{H}}{\lambda_{V}+\lambda_{H}}. \end{array}$$

**Fig 2:**
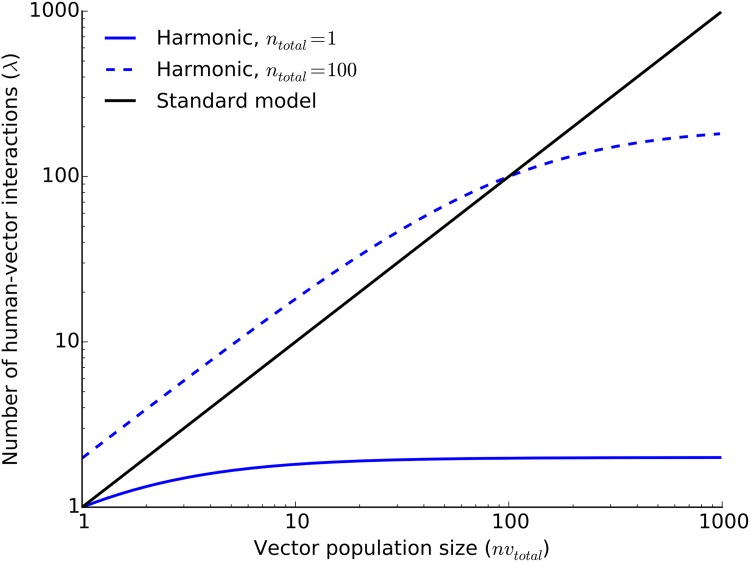
The rate of human-vector interactions. The plot shows the daily average number of human-vector interactions as modelled using the standard approach (*λ*_*V*_, see Methods) or by incorporating humans as a limiting factor (2*λ*_*V*_ *λ*_*H*_/(*λ*_*V*_ + *λ*_*H*_), see Methods) in the presence of 1 and 100 humans.

In the limit *n*_total_ → ∞, *λ* → 2*λ*_*V*_, and in the limit *nv*_total_ → ∞, *λ* → 2*λ*_*H*_, which provides the saturation effect. We note that in the case of the harmonic mean, emerging bite rates are twice the values defined by *b* and *k*_hum_, so parameterisation needs to be fine tuned accordingly. In Fig 2, we demonstrate the behaviour of *λ* compared to the standard model for *b* = *k*_hum_ = 1 and different human population sizes. Evidently, *λ* follows the standard model given the availability of the human host, but a switch in host preference is seen when vectors outnumber humans.

In this context, we employed a fixed human biting rate (*k*_hum_); however, we assumed that the vector biting rate (*b*) is proportional to vector activity, which we linked to the number of eggs laid per day (*F*_4_: fecundity). According to this,

$$\begin{array}{c} \lambda_{V}=k_{\text{vec}}\;F_{4}\;nv_{\text{total}}, \end{array}$$

where *F*_4_ is daily fecundity as simulated by the albopictus model, and *k*_vec_ is a proportionality constant. Incorporating fecundity in vector biting rate helps to establish a closer link between mosquito biting and ovipositioning, which are inherently coupled.

As a result, the daily average number of human-vector interactions can be written as

$$\begin{array}{c} \lambda =\frac{2\;k_{\text{vec}}F_{4}nv_{\text{total}}\times k_{\text{hum}}n_{\text{total}}}{k_{\text{vec}}F_{4}nv_{\text{total}}+k_{\text{hum}}n_{\text{total}}}. \end{array}$$ (2)

Alternative models of human-vector interaction can be used; however, to the best of our knowledge, sufficient empirical evidence is not currently available to critically assess their relative validity. If available, vector biting statistics from human settlements of different sizes, along with the surveillance of local vector populations, could be used for model calibration and validity. Due to its similarity to the standard model when *n*_total_ is large, and the saturation effect it exhibits when *n*_total_ is low, we used Eq (2) to model human-vector interactions.

The fate of each interaction depends on the probability that either one of an interacting pair is infectious. The probability of a vector-to-human transmission can be written as

$$\begin{array}{c} p_{HV}=\frac{nv_{Ai}\;n_{S}}{nv_{\text{total}}\;n_{\text{total}}}\alpha_{\text{scpH}}, \end{array}$$

where *n*_*S*_ is the number of susceptible humans and *α*_scpH_ is the human susceptibility to chikungunya infections. Similarly, the probability of a human-to-vector transmission can be written as

$$\begin{array}{c} p_{VH}=\frac{nv_{As}\;\left(n_{I}+n_{Ic}\right)}{nv_{\text{total}}\;n_{\text{total}}}\alpha_{\text{scpV}}, \end{array}$$

where *α*_scpV_ is the vector susceptibility.

In order to implement the stochastic transmission process, we considered each interaction separately. As stated above, we assumed that the number of daily human-vector interactions is a Poisson-distributed random number with *λ* as the average number of interactions. The probability that any of these interactions results in a transmission is *p*_*HV*_ + *p*_*VH*_, which changes every time a successful transmission takes place. The state transitions associated with each transmission can be written as

$$\begin{array}{ccccc} A_{s} & \to & A_{i} & \text{with} & p=\frac{p_{HV}}{p_{HV}+p_{VH}},\\ S & \to & I & \text{with} & p=\frac{p_{VH}}{p_{HV}+p_{VH}}. \end{array}$$

We note that only a subset of these interactions will likely result in a transmission. Having determined the number of interactions per day, *β*, it is possible to discard the non-transmission interactions by sampling the number of interactions to the next successful transmission event from a geometric distribution (*p* = *p*_*HV*_ + *p*_*VH*_). When the next succesful transmission takes place after *β* interactions, the process stops for the day and continues the next day with another sample of *β*.

In order to improve efficiency in numerical simulations, we approximated the daily number of interactions with a normal distribution with mean and standard deviation *λ* when *λ* is large enough (more than 1000 interactions per day). In this case, the number of interactions to the next successful transmission can be approximated with an exponential distribution with rate *p*_*HV*_ + *p*_*VH*_.

### Spatial spread model

**Fig 3:**
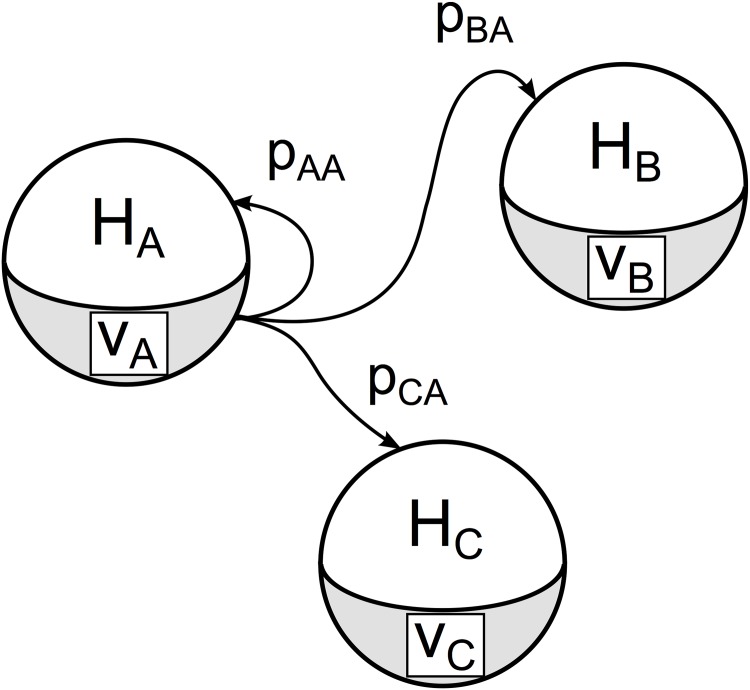
The stochastic metapopulation model of disease spread. Spheres represent populations of humans, *H*, and vectors, *V*. Due to the movement of humans, each vector population is allowed to interact with humans of the same or a different patch with a given probability *p*.

In order to model spread, we implemented a metapopulation model [74, 75] by allocating vector and human populations in patches (Fig 3). Each patch resides in a distinct location and has a size comparable to the maximum flight span of a vector population. We assumed that vector movement between patches due to wind and human activities is negligible; thus, vectors can move within a patch but not between patches. Since only humans are mobile between patches, they are responsible from spreading disease spatially.

While we allowed human mobility, we enforced a fixed population size in each patch. In essence, we allowed humans to interact with the vector populations of neighbouring patches by moving out and back in on a daily basis. We defined *p*_*ij*_ as the probability of the humans in patch *i* interacting with those in patch *j*, or, in case of *i* = *j*, not interacting with any neighbouring patch. By definition,

$$\begin{array}{c} \sum_{j}p_{ij}=1. \end{array}$$

By focusing on the vector population of patch *j*, we calculated the expected number of people in *j* as a result of all population movements,

$$\begin{array}{c} n_{{\text{total}}_{j}}=\sum_{i}p_{ij}H_{i}. \end{array}$$

Similarly,

$$\begin{array}{c} n_{S_{j}}=\sum_{i}p_{ij}n_{S_{i}},\;n_{I_{j}}=\sum_{i}p_{ij}n_{I_{i}},\;\text{and}\;n_{Ic_{j}}=\sum_{i}p_{ij}n_{Ic_{i}}. \end{array}$$

Then, for each bite resulting in disease transmission to a human, we selected the region from where the person originated, *i*, using the weights *p*_*ij*_ *H*_*i*_. In essence, we used *n*_total_*j*__ to estimate the total number of bites in Eq (2), and then the relative sizes and interaction probabilities of each patch to determine the direction of transmission.

## Surveillance datasets

We studied two chikungunya outbreaks: the outbreak in Italy in 2007 and the outbreak on Reunion Island in 2005-2006. In both cases, it was reported that the main vector was the temperate strain of *Ae. albopictus* [4, 9, 76].

According to the reports [7, 9], the outbreak in Ravenna, Italy, took place in the Castiglione di Cervia and Castiglione di Ravenna villages, which accommodate about 4000 people in less than 1 km^2^ area. An infectious human was introduced on the 15^*th*^ of June, 2007, which was followed with 205 confirmed cases of infection in about three months [7]. Since vector control measures were applied from the 18^*th*^ of July [7, 9], we considered only the 110 cases prior to this date for inference. Here, we refer to this surveillance dataset as *δ*_*I*_.

Due to the size of the region, we used a single grid cell with 0.25° resolution and a center at 12.250°E and 44.250°N from the ClimEurope dataset (see Environmental data sets). We extracted daily average temperature (t2m), daily precipitation, photoperiod, and human population density to simulate the expected adult female abundance, *n*_4_, for the grid cell. Using a proportionality constant, *α*_ovt_, we estimated the number of susceptible vectors, *nv*_*As*_, in the area of the outbreak as at least the nearest integer value of

$$\begin{array}{c} nv_{As}=\alpha_{\text{ovt}}\;n_{4}. \end{array}$$

We note that the albopictus model predicts the mean adult vector count per ovitrap and its variability. Since the vector model is deterministic, and the error is small compared to the intrinsic stochasticity of the disease transmission model, we used the mean, *n*_4_, and discarded its variability.

The outbreak on Reunion Island was much larger in comparison, and took place in two episodes: (i) the first started in March 2005 and affected about 3000 people before it settled on a low, but persistent, infection rate from June onwards, (ii) the second episode took place between December 2005 and April 2006 and resulted in about 250,000 infections. In total, about 34% of the entire population of the island (about 780,000 people) was infected [4, 36]. On this basis, we partitioned the cases into two datasets: *δ*_*S*_, which consists of the cases prior to 20 November 2005 [77], and *δ*_*L*_, which consists of all cases from both episodes.

The surface area of the island is approximately 2,500 km^2^, which is close to the size of one grid cell of the ClimGlobe dataset (see Environmental data sets). Therefore, we simulated the expected adult female abundance using the environmental variables from the grid cell centered at 55.625°E and -21.125°N.

We partitioned the island into 238 patches of 9 km^2^ area, each inhabited by at least one person, and assumed that the human population is highly mobile within each patch enabling random interaction with any vector within the patch. We further assumed that the vector population is not uniformly distributed among the patches, but correlates with the human population size in each patch [30]. In essence, we set the number of susceptible vectors in patch *i* as the nearest integer value of

$$\begin{array}{c} nv_{As_{i}}=\alpha_{\text{ovt}}\;n_{4}\;\frac{n_{{\text{total}}_{i}}}{\sum_{i}n_{{\text{total}}_{i}}}. \end{array}$$

We obtained the number of people in each patch from the INSEE gridded population estimate of 2009 [78]. Although the resolution of this dataset is 1 km^2^, we note that the simulation time increases exponentially with the number of patches prohibiting efficient inference and posterior sampling. Therefore, we re-grouped grid cells to yield a computationally feasible number of patches where rapid mixing of humans can still be expected.

**Fig 4:**
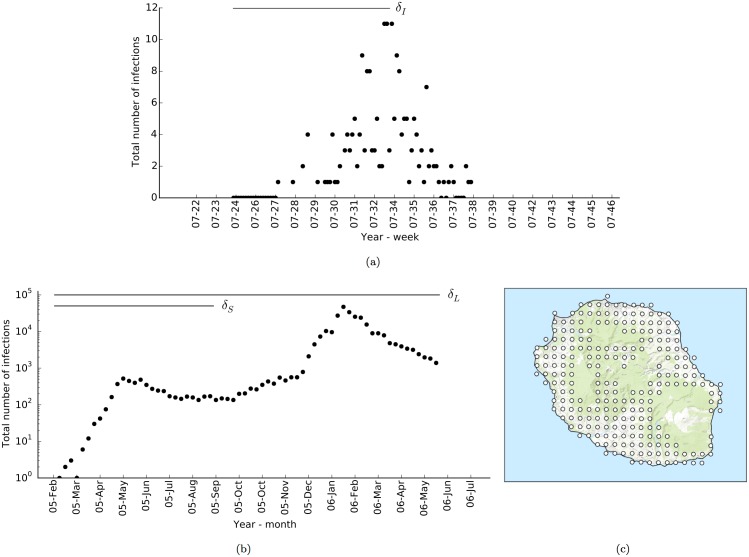
Outbreaks of *Ae. albopictus*-borne chikungunya in Italy and on Reunion Island. (a) Number of reported, and laboratory confirmed, infections in Ravenna, Italy, during the chikungunya outbreak in 2007 [9]. (b) Total number of infections reported by the island-wide surveillance system on Reunion Island during 2005-2006 [36]. The horizontal lines in (a, b) indicate the time frames and infections that each dataset covers. (c) The centroids of the patches of the metapopulation model for Reunion Island.

The cases included in each disease surveillance dataset and the centroids of the patches employed for Reunion Island can be seen in Fig 4. As a first approximation, we assumed that the transfer probability of people between two patches, *p*_*ij*_, is proportional to the inverse of the flight distance between the patches (see Spatial spread model),

$$\begin{array}{c} p_{ij}=\{\begin{array}{cc} q_{i}\;\frac{\sum_{j}d_{ij}^{c}}{d_{ij}^{c}} & \text{if}\;i\neq j\\ 1-q_{i} & \text{if}\;i=j \end{array}, \end{array}$$

where *d*_*ij*_ is the flight distance between patch *i* and *j*, *c* is the degree of the relationship, and *q*_*i*_ is the probability of a person moving away from the patch he or she inhabits. Parameters *q*_*i*_ and *c* together determine the overall mixing rate of the human population on the island, and we included them in the list of parameters to be inferred.

## Results and discussion

### Predicting vector dynamics during the outbreaks

Predicting vector abundance is an essential part of modelling vector-borne disease spread. Most of the previous approaches combined vector dynamics and disease spread modelling [40, 49], where model validation was performed for both in a single step. We argue that, in terms of fitting to outbreak data, this approach gives a greater flexibility to the model and thus might result in overfitting. Unfortunately, vector abundance data are limited or restricted for most parts of the world due to the cost and effort required to collect such data. Here, we employed our environmentally-driven population dynamics model of *Ae. albopictus*, henceforth referred to as the albopictus model [30], which has been validated on the Emilia-Romagna region of Italy [50] and demonstrated reasonably good predictive capacity across Europe.

**Fig 5:**
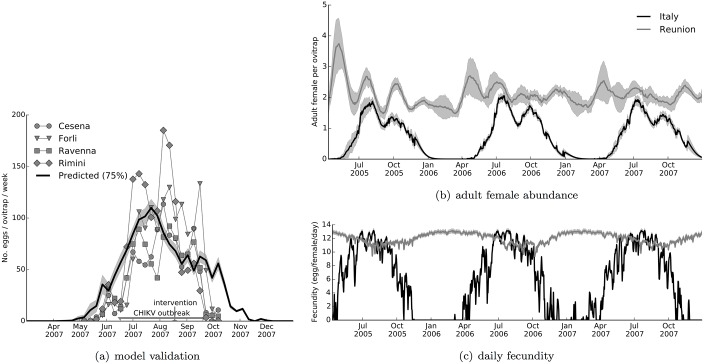
Prediction of adult vector abundance and activity in Italy and on Reunion Island. (a) The predicted number of eggs per ovitrap per week in Castiglione di Cervia and Castiglione di Ravenna villages is shown with the surveillance data from four neighbouring towns [79]: Cesena (circle), Forli (triangle), Ravenna (square), and Rimini (diamond). The beginning and end of the outbreak and the time of application of vector control measures [7, 9] are marked in the figure. The predicted vector abundance is scaled down to 75% in the plot. (b) Predicted adult female vector abundance per ovitrap in the two regions for a time frame covering both the Italian and the Reunion Island outbreaks. (c) Predicted daily fecundity during the same time frame in the two regions. The pale grey shades in (a, b, c) indicate the 95% credible interval of the mean.

Although vector abundance data directly from the two regions of the outbreak were not available, we tested the applicability of the albopictus model over a preliminary surveillance dataset comprising ovitrap samplings in four towns, Ravenna, Forli, Cesena, and Rimini, during the period of the chikungunya outbreak in Italy [79]. We employed the same posterior parameter set Θ4 (see Vector dynamics model) calibrated on the Emilia-Romagna region. In Fig 5(a), we plot model prediction against the average number of eggs collected per ovitrap for each town. The prediction is in agreement with the data with a certain proportionality constant (0.75). Scaling with a proportionality constant was necessary due to the model output being normalised with respect to the number of ovitraps as placed in the Carrieri *et al.* 2011 surveillance study [50].

Although it is possible to improve accuracy by employing high-resolution climate datasets, or meteorological data from local stations, temporally resolved vector surveillance data from the region is not available to improve model calibration. Furthermore, dependence on such extensive resources might render the model highly specific for a given study area and cast limited applicability elsewhere unless such data are available. Therefore, we believe that the level of match in Fig 5(a) permits proceeding with large-scale epidemiological modelling.

While the introduction of *Ae. albopictus* to Italy has been traced to Georgia (USA) through used tyre trade in the early ’90s [80], its establishment on Reunion Island has been known for centuries [81]. Further study by Paupy *et al.* [82] demonstrated a stratified *Ae. albopictus* population structure on the island along the east and west coasts. Nevertheless, Kamgang *et al.* [76] reported close evolutionary relationships between the European and Reunion Island populations. Evolutionary clustering of the Reunion Island *Ae. albopictus* with the temperate strains [83] suggests the applicability of the albopictus model on both regions. Although it is highly unlikely that the same parameterisation will accurately represent both populations, in the absence of vector surveillance data, we believe that the model will be able to capture the essence of environmental dependency of *Ae. albopictus* on the island.

Prompted by this, we used the albopictus model in conjunction with ClimEurope dataset for Italy and ClimGlobe dataset for Reunion Island (see Environmental data sets) to predict adult female vector abundance in the two regions. The expected number of adult female vector per ovitrap (ovitraps as placed in the original surveillance study) and the predicted daily fecundity are given in Fig 5, respectively in Fig 5(b) and 5(c). As shown, seasonality in vector abundance and activity are more evident in Italy where the model predicted no significant presence during winter. However, abundance and egg-laying activity fluctuate at high levels all year round on Reunion Island. Uninterrupted annual activity is also supported by the incidence data from the island during the time between the two outbreaks when around 100 infections continued to be observed weekly (Fig 4).

### Modelling the chikungunya outbreak in Italy

We adopted a Bayesian approach to investigate the degree of support each outbreak surveillance dataset (see Surveillance datasets) offers to the model. Restrictions imposed by the data on the posterior probability distribution can be used to evaluate model assumptions and compare epidemiological trends in different outbreaks. According to Bayes’ theorem, the posterior probability of *θ*, a vector with elements corresponding to each model parameter, is proportional to the likelihood, Pr(*δ*|*θ*), and the prior probability, Pr(*θ*),

$$\begin{array}{c} \mathrm{Pr}(\theta |\delta )\propto \mathrm{Pr}(\delta |\theta )\mathrm{Pr}(\theta ). \end{array}$$

The prior probability is defined in reference to the literature and expert knowledge. Given the degree of uncertainty in chikungunya epidemiology, we imposed only weak restrictions and defined sufficiently wide intervals for all model parameters (Table 1) within which the prior probability is uniform. The likelihood is defined as the probability of replicating the data with the model with a given parameter configuration. When the likelihood cannot be determined, or is computationally intractable, it is possible to apply approximate Bayesian computation (ABC) and replace it with a simulation-based comparison of model output and observation [84–86]. According to this, an arbitrary distance function is defined, *d*(*y*, *δ*), to inform when model output, *y*, is sufficiently close to the data. The likelihood is then replaced with an approximate probability based on this distance and a threshold value, *τ*. When *τ* is sufficiently small, better estimates of posterior probability can be obtained.

It is often computationally unfeasible to have a small enough threshold. This would prohibitively decrease the acceptance rate in Markov chain Monte Carlo (MCMC) or sequential Monte Carlo (SMC) algorithms. Here, instead of a binary in-or-out comparison, we used a probability function proportional to the *χ*^2^ distance between simulated and observed data,

$$\begin{array}{c} \mathrm{Pr}\left(\theta |d\left(y,\delta ,\tau \right)\right)=C\prod_{i}\exp\{-\frac{1}{2}\frac{{\left(\delta_{i}-y_{i}\right)}^{2}}{\sigma_{i}^{2}}\}, \end{array}$$

where *C* is the normalisation factor, *δ*_*i*_ is the case report at the time corresponding to the *i*^*th*^ data point, and *y*_*i*_ is the corresponding random sample from the model with parameter *θ*. We defined the variance $\sigma_{i}^{2}$ as a function of *τ* and *δ*_*i*_,

$$\begin{array}{c} \sigma_{i}^{2}=\{\begin{array}{cc} \tau \delta_{i} & \text{if}\;\delta_{i}\geq 1\\ \tau & \text{otherwise} \end{array}. \end{array}$$

With this definition, we anticipated smaller certainty on high infection counts and greater certainty on low infection counts.

We note that, here, *τ* is a variable for increasing or decreasing the acceptance probability. It is equivalent to the annealing temperature of simulated annealing [87], or the hoppMCMC algorithm [88], or to the acceptance threshold of approximate Bayesian computation. The equation generates an energy gradient, with a minimum at *y* = *δ*, which could be traversed with an MCMC algorithm. When compared with traditional ABC, the gradient distance function yields better posterior samples by assigning more weight to samples from the vicinity of *δ* [85].

Although ABC algorithms have improved in efficiency [89, 90], Bayesian methods are inherently limited in capacity when large systems with many parameters are of concern [91]. In order to circumvent this limitation, we assumed that the posterior distribution can be approximated as a combination of a finite number of Gaussian distributions with modes corresponding to each of its local maxima. Following the previous definition of this concept in Erguler *et al.* 2016 [30], we refer to the locality of each of these Gaussians as a posterior mode.

We note that model dynamics will change as parameter values change; however, the locality of a given parameter configuration is associated with similar behaviour. The extent of this locality depends on sensitivity at the specific region of the posterior distribution [92–95]. Therefore, sampling from within a posterior mode will likely yield a set of *θ* associated with similar model behaviour.

When posterior modes are sufficiently distant from one another, low-probability regions of the posterior distribution prevent efficient sampling. Here, instead of sampling from the entire posterior distribution, we opted to sample from the posterior modes with the highest average probabilities. Our inference procedure comprised of two distinct steps: (i) identifying a high-probability region in the posterior distribution with an optimisation step, and (ii) obtaining posterior samples within this region with a sampling step.

We used the python (v2.7) implementation of the hoppMCMC (v0.5) algorithm, which benefits from a variable acceptance probability to jump between posterior modes and arrive at the ones with high-probabilities. We provided the probability function Pr(*θ*|*d*(*y*, *δ*, *τ*)) to the algorithm using the term *τ* as the annealing temperature, and explored high-probability posterior modes within the parameter domains given in Table 1. Once identified, we sampled from the posterior mode using the adaptive Gibbs sampling algorithm of the hoppMCMC package. We initiated each sampling step with the optimum parameter configuration identified in each optimisation step. We ran 50 chains for 2 × 10^4^ iterations, discarded the initial half, and extracted 20 parameter configurations from each chain with regular intervals. We monitored the chains and their autocorrelation to ensure convergence (see S1 File for detailed methodology). We note that our choice of hoppMCMC is motivated by its performance and efficiency for systems with large number of parameters. If found wanting, a combination of alternative optimisation and Bayesian sampling algorithms can be used.

As a result, we identified a single posterior mode, labelled *Q*_*I*_, using the dataset *δ*_*I*_ for Italy. We sampled 1000 parameter vectors from *Q*_*I*_, and simulated an epidemic trajectory with each of them. The emerging set of epidemic trajectories is

$$\begin{array}{c} y=\{x|x∼\eta \left(\theta \right)\;\text{and}\;\theta ∼Q_{I}\}. \end{array}$$

It is important to note that the variability in *y* is a result of both parametric variation within the posterior mode and also intrinsic stochastic variation. In order to facilitate effective sampling and to maintain a non-prohibitive acceptance rate, around 1%, we set *τ* = 2.

**Fig 6:**
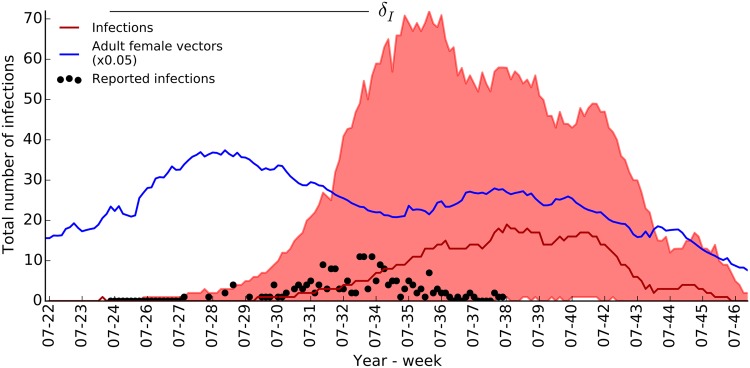
Epidemic trajectory of the Italian CHIKV outbreak as predicted with posterior mode *Q*_*I*_. Median, red line, and 95% range, red shade, of epidemic trajectories are plotted together with adult abundance, blue line, and incidence reports, dark circles [7, 9].

The predicted epidemic trajectory is given in Fig 6 with the 95% range and the median of reported symptomatic infectious cases. For clarity in plotting, we only consider the simulations where the index case (15^*th*^ of June, 2007) resulted in a secondary infection.

The model predicts that the outbreak could have lasted longer if not for the administration of effective vector control measures [7, 9]. According to the model, the probability for a secondary infection resulting from the imported (index) case was as high as 0.43. In addition, between the time of virus introduction and the predicted end of the outbreak, towards the end of November, the model predicted that the median number of human infections, including both symptomatic and asymptomatic cases, was 1700 (95% CI: 100—3900). These predictions correspond to a high outbreak risk for the combination of location and time of virus introduction. We estimated both the probability and impact of the outbreak to be in agreement with the data and the previous study of Poletti *et al.* [40]. We note that our inference procedure employed no prior information on parameter values, but benefitted only from the observed epidemic trajectory. The degree of uncertainty can be reduced if previously reported restrictions on parameters were imposed; however, we aimed to demonstrate the effectiveness of the described methodology for developing reliable and predictive models of *Ae. albopictus*-borne diseases for which little or no experimental studies are available.

We assessed outbreak severity with two indicators: the outbreak risk and impact. We defined the outbreak risk as the probability that an infected host gives rise to a secondary infection in a fully susceptible human population. The outbreak impact is, on the other hand, the number of human infections observed during the course of an outbreak. We calculated these indicators numerically from the model output, *y*, and compared them with the common indicators *R*_0_ and *p*_0_. *R*_0_ is an estimate for the number of secondary infections arisen by the introduction of a single infected host to a fully susceptible population, while *p*_0_ is the probability of an outbreak as defined for SEI-SEIR models [40, 61, 71, 73]. The equations for *R*_0_ and *p*_0_ are

$$\begin{array}{ccc} R_{VH} & = & b\times \frac{\alpha_{\text{scpH}}}{\mu_{v}},\\ R_{HV} & = & b\times \alpha_{\text{scpV}}\times \gamma \times \frac{nv_{\text{total}}}{n_{\text{total}}}\times \frac{1}{1+\omega_{\nu }\mu_{v}},\\ R_{0} & = & R_{VH}R_{HV},\;\text{and}\\ p_{0} & = & 1-\frac{R_{VH}+1}{R_{VH}\left(R_{HV}+1\right)}, \end{array}$$

where *b* is fixed vector biting rate and *μ*_*v*_ is adult vector mortality. In order to compare the two approaches, we employed literature-based parameter values—as listed in Poletti *et al.* 2011 [40]—for *R*_0_ and *p*_0_: *b* = 0.25, *ω*_*ν*_ = 2 to 4 days, *γ* = 2 to 7 days, *α*_scpH_ = 0.5 to 0.8, *α*_scpV_ = 0.7 to 1. Where a range is defined instead of a point estimate, we treated the value as a uniform random variable within the defined range. We estimated the average adult vector count and mortality for each time point from the albopictus model. We used the average lifespan, *μ*, inferred in the Vector dynamics model section to estimate mortality, *μ*_*v*_ = 1 − 1/*μ*. Since the albopictus model predicts vector abundance with regard to the ovitrap density in the original surveillance study [50], we scaled the abundance to match the levels reported in Poletti *et al.* 2011 [40] (*nv*_total_ = 1.4 × 10^5^ *n*_4_).

**Fig 7:**
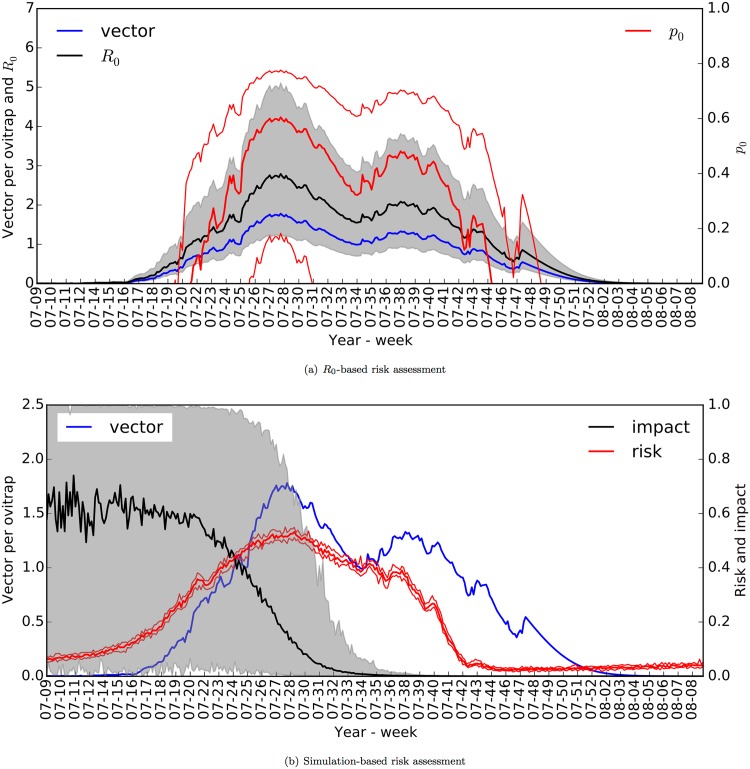
Risk assessment of *Ae. albopictus*-borne chikungunya. In (a), *R*_0_ (black line and grey shade, scale on the left) and *p*_0_ (red lines, scale on the right) are plotted together with *n*_4_, the expected adult vector count per ovitrap from the albopictus model (blue line, scale on the left). In (b), the outbreak risk (red lines, scale on the right) and impact ratio (fraction of total infections, black line and grey shade, scale on the right) are plotted together with *n*_4_ (blue line, scale on the left). Thick and thin lines (or shade) indicate the median and the 95% range.

In Fig 7, we demonstrate the behaviour of each indicator with respect to the time of introduction of the index case. We found that both *R*_0_ and *p*_0_ follow vector abundance closely, with the exception that *p*_0_ drops sharply at the beginning and end of the mosquito season—delineated by the initial increase and final decrease in vector abundance (Fig 7(a)). In Fig 7(b), we found that the outbreak risk is higher at the beginning of the mosquito season, then it drops gradually. The risk decreases earlier than does *p*_0_ as it is associated with both vector count and activity.

The non-zero outbreak risk is due to the existence of the chronic stage, often overlooked by traditional modelling approaches; the introduction of an infectious person might trigger a chronic infection, which might relapse anytime during a high-risk period. According to the mission report of the European Centre for Disease Prevention and Control (ECDC) [7], the index case was introduced on 15 June, but the infectious episode resulting in the outbreak was a relapse on 23 June, which makes the modelling of the chronic stage relevant for the Italian chikungunya outbreak.

While *R*_0_ and *p*_0_ pertain to the outbreak risk, the impact of a possible outbreak can be determined by following transmission dynamics in time. The model suggests that the impact is larger if an outbreak takes place at the beginning of the mosquito season (Fig 7(b)). In essence, time-dependent risk assessment of an *Ae. albopictus*-borne chikungunya outbreak can be categorised into four consecutive stages: low risk-high impact, high risk-high impact, high risk-low impact, and low risk-low impact.

Since there is a high outbreak risk in the area and the impact of a possible outbreak can be very high, the importance of vector control measures in containing large-scale epidemics is evident. According to the mission report of ECDC/WHO [7] and Rezza *et al.* 2007 [9], a series of vector control measures, including egg/breeding site removal and larvicide/adulticide treatment, was applied in the region for 3 consecutive days commencing on the 18^*th*^ of August. The estimated impact was more than 90% reduction in adult mosquitoes, about 60% reduction in eggs and breeding sites, and about 95% reduction in larvae and pupae [40]. We mimicked the potential effects of this intervention by applying 60% daily reduction in adult survival for 3 days, 25% daily reduction in eggs and breeding sites for 3 days, and 10% daily reduction in larvae and pupae for 30 days, the expected duration of larvicide activity [40].

**Fig 8:**
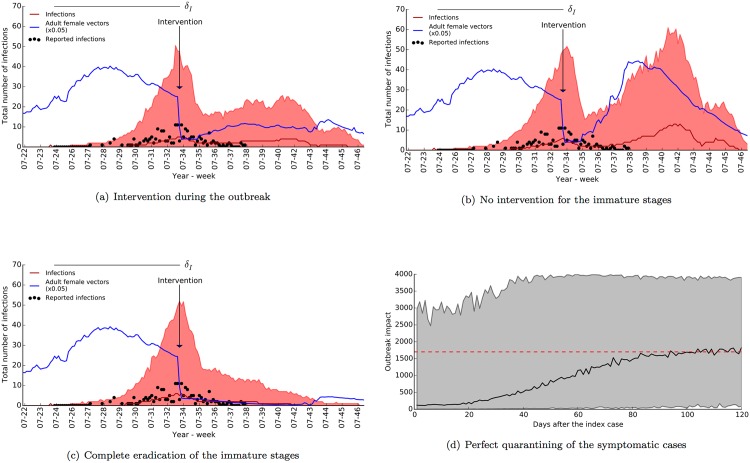
Outbreak management strategies for Italy. Median, red line, and 95% range, red shade, of epidemic trajectories are plotted together with adult abundance, blue line, and incidence reports, dark circles [7, 9]. The vector control measures reported in the literature [7, 9, 40] were implemented in (a). Only adulticide treatment was implemented in (b). A stronger control strategy on the immature stages and breeding sites was implemented in (c). The effect of perfectly isolating the symptomatic cases is shown in (d) with a black line, median, and a grey shade, 95% range. The red dashed horizontal line indicate the median outbreak impact (1700 human infections) without intervention.

As seen in Fig 8(a), the intervention drastically reduced the number of adult vectors and maintained a low transmission rate up to the end of November (470 cases instead of 1700). The initial decline due to the effective reduction of adult vectors provided a short-term high-efficiency control, while breeding site removal and control of the immature stages maintained the long-term effectiveness of the intervention. Despite high parametric and intrinsic variability, the model agrees well with the previous study of Poletti *et al.* [40] in terms of the effects of vector control. Further to the applied control measures, we investigated the possible impacts of alternative strategies. For instance, rapid recovery of high transmission rate is evident in Fig 8(b) where only adulticides were applied (900 cases instead of 1700). The strong suppression due to complete eradication of breeding sites and the immature stages is apparent in Fig 8(c); assuming 100% reduction achieved during the time frames of the vector control measures (310 cases instead of 1700).

We also tested the effect of quarantining humans by asserting a hypothetical scenario where symptomatic individuals—the ones detected following implementation of the strategy—are completely isolated from the vector population. Despite high variability, we identified that the median number of cases decreases substantially if the intervention is implemented promptly (Fig 8(d)). The model suggests that quarantining maintains its effectiveness if implemented within 20 days of the index case.

**Fig 9:**
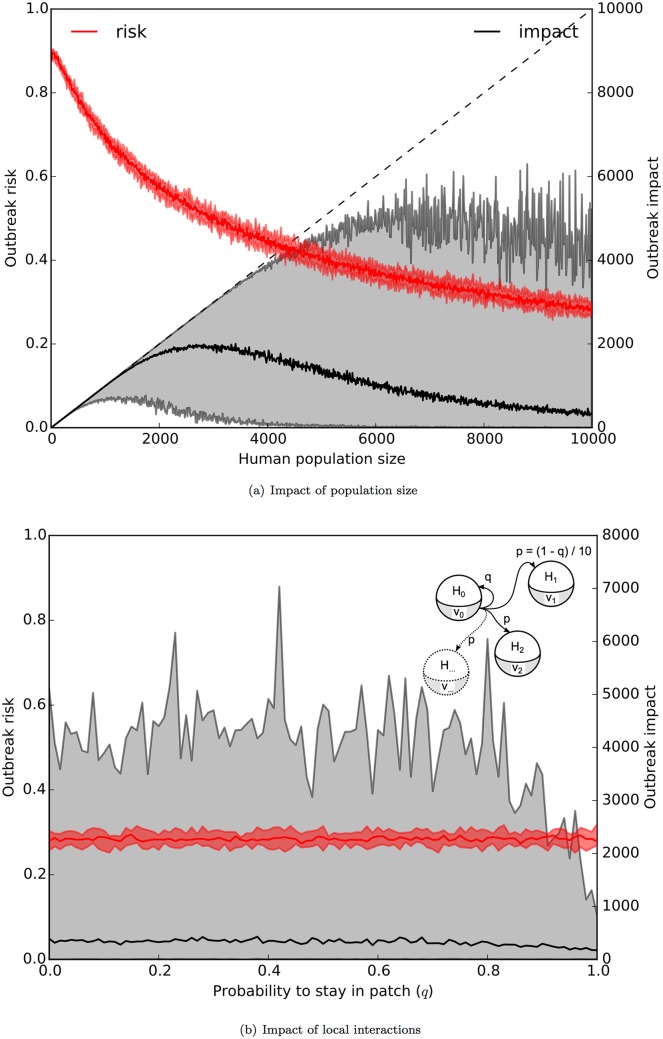
Effect of population size and stratification. In (a), outbreak risk (red, left axis) and impact (black, right axis) are plotted with respect to human population size. In (b), outbreak risk (red, left axis) and impact (black, right axis) are plotted with respect to the connectivity, *q*, of 10000 people equally distributed in 10 patches (see text). Solid lines indicate median and shades indicate 95% range.

In addition to quarantining, which focuses only on detectable symptomatic cases, we investigated the effect of isolating a part of the human population from the vector population. Potential means of isolation could include using mosquito repellants or imposing travel restrictions. We assessed the outbreak risk and impact by varying the human population size for the Italian CHIKV outbreak (Fig 9(a)). We found that increasing population size decreases the outbreak risk as it is less likely for an infectious person to be bitten in a large population. The impact of an outbreak is small, but less variable, in small populations. Although almost everyone will get infected, isolation effectively prevents transmission to a larger population where the impact would be greater. We observed that the impact does not follow population size linearly, but tends to decrease as size increases. This is due to the speed of transmission: as the progression is slower in large populations, it takes multiple mosquito seasons for the infection to spread through the population. Here, we followed transmission for a year following the last reported case.

For large populations scattered over large areas, the assumption of rapid mixing might not be valid. We tested the effect of population stratification by partitioning both the human and the vector populations into 10 identical interconnected patches. We rendered the patches equidistant from each other, and tuned human mobility using *q*, the daily probability of residing in a patch (Table 1). We found that the outbreak risk is insensitive to *q*, as the vector to human ratio is the same in both a single patch or all of them combined (Fig 9(b)). However, the impact, more specifically the possible range of the impact, decreases substantially when the human population is confined to reside in patches (when *q* is close to 1).

Overall, we argue that restricting human mobility is an effective outbreak management strategy, especially in large populations; however, given the impracticality and the economic burden of imposing such a restriction, we suggest that the combination of quarantining symptomatic cases and implementing various vector control strategies could help to contain large outbreaks effectively.

**Fig 10:**
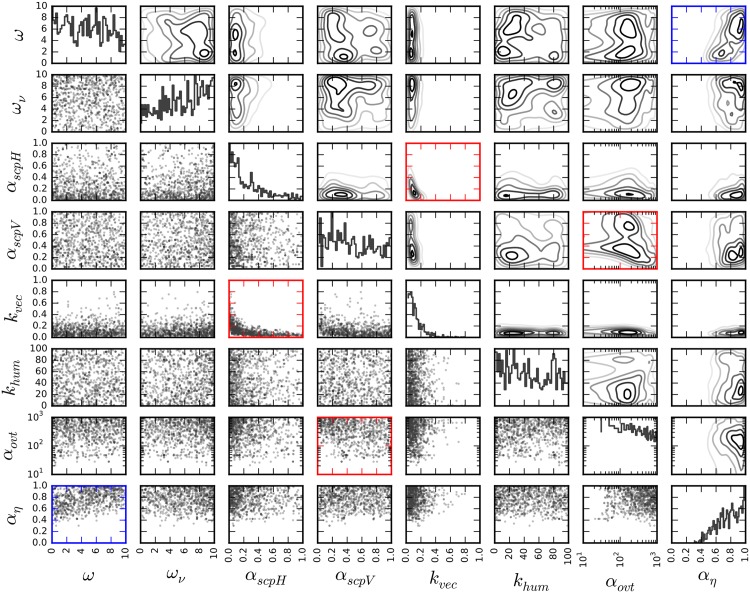
Marginal probability distribution of posterior mode *Q*_*I*_. Marginal posterior distributions with 1000 samples from *Q*_*I*_ are shown. Gaussian kernel density estimates are plotted in the upper triangular, marginal probability distributions are plotted on the diagonal, and the samples are shown in the lower triangular. Strong (*ρ* < −0.25 and *ρ* > 0.25) and statistically significant (*p* ≪ 0.001) correlations were indicated with red and blue frames for negative and postitive correlations, respectively.

Following the analysis of model predictions, we investigated the information acquired from the surveillance dataset as a result of the inference process. In Fig 10, we present the marginal probability distributions of posterior mode *Q*_*I*_ for a subset of parameters (see S1 Fig for the full list of parameters). As evident from the figure, vector biting rate, *k*_vec_, and human susceptibility, *α*_scpH_, are likely to be low, but vector counts, imposed by *α*_ovt_, and reporting rate, *α*_*η*_, tend to be high with regards to their respective search domains. Interdependencies between parameters, evident in the posterior mode, indicate that parameter variability allows maintaining a reservoir of infected vectors and a non-zero biting rate for the duration of the outbreak. Strong negative correlation (Pearsons’ *ρ* < −0.25 and *ρ* > 0.25 with *p* ≪ 0.001) between *k*_vec_ and *α*_scpH_ and between *α*_ovt_ and *α*_scpV_ suggests that a surge of biting rate, or equivalently vector count, is counterbalanced by a reduction of susceptibility in order to match the observed epidemic trajectory. We observed that it is almost twice as likely for a chronic infection to relapse as to transcend to the recovered stage, evident from the joint distribution of *p*_*CI*_ and *p*_*CR*_. However, the variability in *p*_*C*_ indicates that the chronic stage has only a minor impact on the course of an outbreak according to the model and *δ*_*I*_. We detected a significant positive correlation between reporting rate and latent period in humans, *ω*, which indicates that a reduction in the fraction of asymptomatic cases would be counterbalanced with a rapid incubation period to match the observed number of cases.

According to the literature, latent and infectious stages do not typically last for more than a week [38–41, 43], while human—based on Reunion Island population [38]—and vector [44] susceptibilities were estimated to be higher than 50%. Although the posterior mode *Q*_*I*_ covers a wide range of biologically plausible parameter configurations, the dataset *δ*_*I*_ does not impose strong restrictions on most of the parameters.

A notable exception is that human susceptibility is about four times more likely to be below 50% according to the dataset. We found that a higher biting rate could alleviate this restriction and allow for higher susceptibility; however, the typical ovipositioning interval of four days [22] does not permit high biting rates. Low human susceptibility might be an inherent characteristic of the human population in the region, or a phenotype of the specific CHIKV strain that infected the area. In addition, it is likely that factors other than what we have accounted for in the model could play significant roles. For instance, the frequency of human-vector encounters depends on the time of the day and whether a person is indoors or outdoors, and this might reduce the estimated number of daily bites through the reduction of *α*_scpH_ and *k*_vec_.

In order to assess the relative impact of parameters to the course of the outbreak, we performed sensitivity analysis by approximating the posterior mode with a multivariate Gaussian distribution [30, 92], and normalising with the sizes of the search domains, [*θ*] (see Table 1),

$$\begin{array}{c} H=\text{diag}{\left(\left[\theta \right]\right)}^{T}\;\Sigma^{-1}\;\text{diag}\left(\left[\theta \right]\right), \end{array}$$

where diag indicates a diagonal matrix of [*θ*], ^*T*^ refers to matrix transpose, and Σ^−1^ is the inverse of the covariance matrix. According to this, parameter sensitivity for *θ* is the corresponding diagonal element of H, *i.e.* $H_{\theta }$.

Sensitivity analysis identified *k*_vec_ as the highest impact parameter, approximately three times more potent in changing model output than the following, *α*_*η*_. Parameters directly controlling susceptibility and vector abundance, *α*_scpH_, *α*_scpV_, and *α*_ovt_, also have high sensitivities, which suggests that vector activity, biting rate, has a pivotal role in determining the rate and impact of disease spread (see S2 Fig). Due to their high sensitivities, and as a consequence of Bayesian inference, these parameters might bear the indirect effects of some of the factors which we have not accounted for, *e.g.* variability of vector and human activities during the day.

### Modelling the chikungunya outbreak in Reunion Island

Motivated by broad applicability of the albopictus model, demonstrated in Europe, and the success of the integrated disease transmission model shown above, we studied the Reunion Island chikungunya outbreak in 2005-2006.

Due to the lack of information on the coverage and effectiveness of the vector control measures [38, 96] applied during the second, larger, incidence peak in 2006, we considered the scenario with no intervention. Accordingly, we predicted that vector abundance and fecundity, despite fluctuating significantly, would have maintained their levels throughout the year (Fig 5).

**Fig 11:**
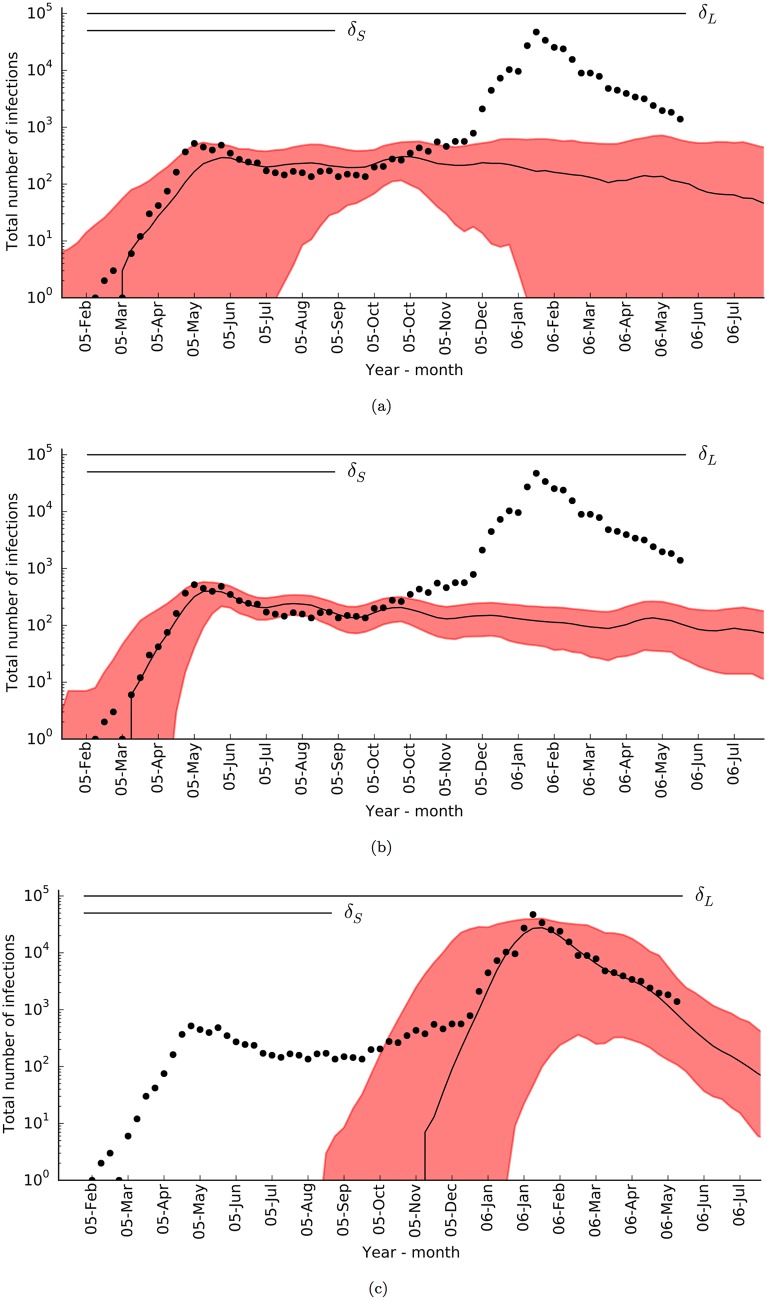
Epidemic trajectory of the Reunion Island chikungunya outbreak as predicted with *Q*_*C*_ (a), *Q*_*S*_ (b), and *Q*_*L*_ (c). Median, black line, and 95% range, red shade, of the trajectories provided that a secondary transmission occurred, plotted in logarithmic scale together with incidence reports [4, 36], black circles. Time spans of the surveillance datasets, *δ*_*S*_ and *δ*_*L*_, are shown on top of each plot.

As a result of the inference employing either of the disease surveillance datasets, *δ*_*S*_ or *δ*_*L*_, we arrived at three characteristic posterior modes: *Q*_*C*_ with *δ*_*S*_ and *τ* = 50, *Q*_*S*_ with *δ*_*S*_ and *τ* = 50, and *Q*_*L*_ with *δ*_*L*_ and *τ* = 1000 as shown in Fig 11. Similarly to the case of Italy, *τ* was chosen to maintain an acceptance rate of 1% in posterior sampling. As a result, posterior modes *Q*_*C*_ and *Q*_*S*_ described the initial smaller outbreak well, but could not capture the second larger peak. Posterior mode *Q*_*L*_ fitted well to the large peak, although with considerable variation, and missed the initial small peak. The inference process did not yield a posterior mode to explain both peaks of incidence (see Fig 4).

Paquet *et al.* [77] argued that the outbreak disseminated as clusters affecting one town after another during the progression of the initial peak. Although posterior mode *Q*_*C*_ predicted a localised spread, which started from the north of the island and progressed towards south along the east and west coasts, the spread took place during both the small and large peaks (see S3 Fig). Conversely, posterior modes *Q*_*S*_ and *Q*_*L*_ suggest that the virus quickly spread over the island. In the case of *Q*_*S*_, the outbreak affected almost half the population in 2005 (95% CI: 20%-75%) and the rest in 2006 (see Fig 11(b)), while in the case of *Q*_*L*_, the outbreak affected almost the entire human population of the island (95% CI: 95%-100%) during the large peak (see Fig 11(c)).

All three posterior modes suggest that reporting rate, *α*_*η*_, was lower than 40%, and eventually, almost everyone on the island was eventually infected. High attack rate necessitates that the fraction of asymptomatic cases is much larger than previously estimated. Seroprevalence studies estimated that about 40% of the population developed antibodies against chikungunya virus [4, 97], which suggests that the majority of infections were symptomatic and captured effectively by the surveillance system. According to this, the virus spread across the entire island as early as the first peak, but affected only a small fraction of the population before it escalated.

We note that some previous modelling approaches, by assuming no environmental dependence in vector populations, fitted only to the first [38] or second peak [49]. The bimodal nature of the outbreak was captured by two studies: Bacaër, by assuming a hypothetical periodic vector abundance [96], and Dommar *et al.*, although only a qualitative fit was reported, by assuming rainfall dependence [98].

Although a posterior mode to explain both peaks and the spread of the virus accurately could be hidden in the parameter space, according to the identified posterior modes, it is likely that one or more of the assumptions with the albopictus model or epidemiological model requires refinement. A possible issue is the assumption of rapid mixing by the human population. The impact of intricate local interactions between the human and vector populations on Reunion Island might be too complex to be represented by a set of 9 km^2^ patches separated by Euclidean distances. By incorporating traffic routes and demographic information, such as large corporate hubs where people from different parts of the island meet regularly, might improve prediction of the rate and routes of the spread.

While we adopted a large-scale modelling approach and assumed that the distribution of the vector population is uniform throughout the island, it is highly unlikely that the microclimatic conditions are similar throughout. In addition, the bimodal nature of the outbreak suggests that either vector abundance or activity might overlap with the two peaks. The albopictus model predicted that vector activity agrees with the peaks, but abundance is more erratic (Fig 5). In addition, the albopictus model has been shown to rely more on temperature and photoperiod [30], but less on rainfall.

Predictions could improve with a finer resolution of environmental variables, or with data from local weather stations; however, vector surveillance data comprising adult abundance from various parts of the island are required to accurately calibrate the model for the island. With such data, it could be possible to assess the rainfall dependence of the island’s vector population in comparison to Italy. Developing an island-specific outbreak model is beyond the scope of this research, but we argue that the observed performance forms a strong foundation for future development of such a model.

As an alternative, a low reporting rate and the lack of a posterior mode fitting both peaks at the same time might be an indication of the involvement of more than one viral strain, and thus multiple outbreaks with overlapping time frames in the region. We countenance the potential scenario where a weak strain of chikungunya virus spreads across the island in accordance with *Q*_*S*_, and acquires one or more adaptations to initiate a secondary wave of infection, which manifests itself as the second larger peak of the outbreak. Experimental evidence reported by Vazeille *et al.* supports this scenario with the identification of two CHIKV strains, at different times, with considerably different transmission capabilities [99].

**Fig 12:**
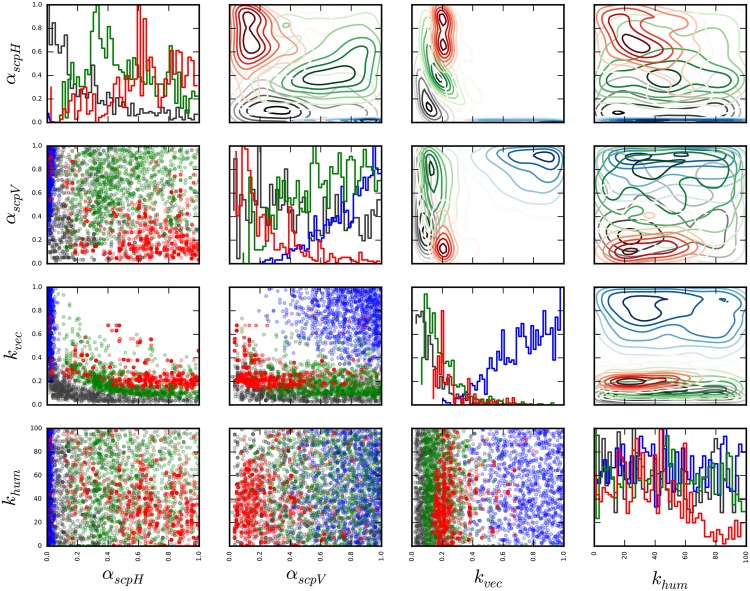
Marginal probability distributions of posterior modes *Q*_*I*_, *Q*_*S*_, *Q*_*L*_, and *Q*_*C*_ for a subset of parameters. Marginal posterior distributions with 1000 samples from each posterior mode are shown for *α*_scpH_, *α*_scpV_, *k*_vec_, and *k*_hum_. Gaussian kernel density estimates are plotted in the upper triangular, marginal probability distributions are plotted on the diagonal, and the samples are shown in the lower triangular. *Q*_*I*_: black, *Q*_*S*_: blue, *Q*_*L*_: green, and *Q*_*C*_: red.

Fig 12 demonstrates the similarity of posterior modes *Q*_*I*_ and *Q*_*L*_, and *Q*_*C*_ to a smaller extent, as opposed to *Q*_*S*_ (see S4 Fig for the full list of parameters). Overall, *O*_*I*_ appears to overlap more with *Q*_*L*_ compared to *Q*_*S*_ and *Q*_*C*_, which could be an indication of a possible common origin between *O*_*I*_ and *Q*_*L*_. Finally, the posterior distribution for a slow localised spread, *Q*_*C*_, is confined to a smaller space compared to the other posterior modes, which demonstrates the strict conditions necessary to match the observed number of cases with this scenario. An island-specific model is, thus, necessary to improve the accuracy of predictions, which is the subject of future research.

## Conclusion

We developed a stochastic spatiotemporal disease transmission model for *Ae. albopictus*-borne chikungunya, and adopted a large-scale modelling approach whereby globally available gridded environmental datasets drive the dynamics of vector populations. This permits a unified framework to examine outbreaks in territories with reasonably comparable environmental features. The ability to use general circulation models as a source of climate data permits prediction of disease epidemiology in the recent past, present, and future. We developed a generic modelling strategy where surveillance datasets inform the *Ae. albopictus*-borne disease tranmission model in the absence of costly and laborious experimental studies. Our analyses suggest that the strategy is applicable over the areas where the albopictus model is applicable, and it can be used to compare and contrast different outbreak scenarios.

We aimed to improve model structure and relax some of the simplifying assumptions in traditional modelling approaches, such as deterministic dynamics, unrealistic biting rates, and the omission of infection relapses. We modified the dynamics of human-vector interactions by introducing vector activity as an additional influence. We presented numerically-estimated outbreak risk and impact as preferable means of risk assessment, and demonstrated that the approach makes better use of the known biology to the extent that it is incorporated into the model. Instead of imposing previously identified restrictions and estimates on certain parameter values, we sought to have the values informed by surveillance datasets. Although proving the validity of these improvements is not a trivial task, we believe that the model we have developed can be a useful tool to help improve understanding of the underlying biology especially when more detailed vector and disease surveillance data are available.

On account of the evolutionary proximity of the *Ae. albopictus* populations on Reunion Island and in Italy, we examined the two outbreaks that took place in these areas during 2005-2006 and in 2007, respectively. To the best of our knowledge, this is the first study to combine the two outbreaks in a unified environmentally-driven modelling framework.

As a result, the model successfully replicates the case reports from Italy, and also informs about the possible effects of various outbreak management strategies. Although the model demonstrates high variability, which should be expected in such relatively small-scale outbreaks, it suggests a non-zero outbreak probability for outside the mosquito season by taking into account possible infection relapses. We identified that the outbreak risk increases gradually, peaks around July, and decreases rapidly during October when the temperatures are favourable for adult vector survival, but not for frequent ovipositioning. By assessing both the potential impact and probability of an outbreak, we suggest that the highest threat to public health from *Ae. albopictus*-borne chikungunya in the region is from May to August.

We identified two strategies of vector control, targeting adults and immature stages/breeding sites, as effective vector control measures only when applied in combination. We found that targeting adult vectors has a short-term impact, but is also highly effective in reducing the transmission rate. Without effective control of the immature stages and removal of breeding sites, this suppression tends to quickly lose effectiveness and restore the initially fast transmission rate in the long run. We reassert the importance of rapid implementation of management strategies, especially quarantining to improve the overall effectiveness. We suggest that unspecific restrictions on human travel to and from affected areas should be considered as an effective strategy, especially in large-scale epidemics.

When applied to the two-stage chikungunya outbreak on Reunion Island, the model demonstrates that a single parameter configuration, or a single posterior mode, is not sufficient to fit to the bimodal nature of the outbreak. While the posterior mode best explaining the first peak is characterised by low human susceptibility and high vector activity, the posterior mode for the second peak is characterised by high human susceptibility and low vector activity. An alternative posterior mode imposing a slow localised spread is also insufficient in describing the timing and distribution of the infected cases.

Although it has been reported that temperate strains of *Ae. albopictus* inhabit both Italy and Reunion Island, our analysis suggests that the environmental dependence captured by our vector dynamics model, assuming identical environmental dependencies in the two locations, might not be applicable to the island’s vector population. As an alternative explanation, we argue that multiple viral strains might have resulted in a two-stage outbreak with considerable epidemiological differences, such as human susceptibility to the infection.

We argue that the performance of the model in Italy promotes its applicability in Europe; however, more data are required to validate its applicability on Reunion Island. An island-specific model requires vector surveillance data from across the island to calibrate the *Ae. albopictus* population dynamics model. In combination with a higher resolution of environmental variables, or direct use of local meteorological data, this will improve vector abundance predictions and reduce the systematic error propagating to the disease transmission model. Subsequently, geo-referenced incidence data, marking the times and locations of the cases, are needed, along with a detailed assessment of the impact of vector control measures, to improve parameter inference for the disease transmission model.

We argue that the model is useful in identifying gaps in our knowledge on global outbreaks. Discrepancies between data and predictions point to biologically relevant assumptions, which could be improved upon closer investigation of the case and accumulation of data. As we improve the model, we develop a better understanding of the insect biology, its environmental dependence, and the global dynamics of *Ae. albopictus*-borne chikungunya outbreaks. The current study is a step towards a global outbreak model for *Ae. albopictus*-borne chikungunya, and a foundation for developing widely applicable disease transmission models for other vector-borne diseases.

## Supporting information

S1 Text Age-dependent survival. Derivation of daily survival probability from gamma-distributed survival time.
(PDF)

S2 Text Posterior mode Θ4 for *Aedes albopictus*. A brief analysis of the albopictus model posterior mode Θ4.
(PDF)

S1 File Posterior sampling. This archive contains the data and the python code required to run the model and perform parameter inference. The archive contains four files. The data files dataItaly.json and dataReunion.json hold environmental variables and surveillance datasets for each region. The python scripts runItaly.py and runReunion.py describe the steps needed to run simulations and perform parameter inference for each region.
(ZIP)

S1 Fig Posterior mode *Q*_*I*_. Pairwise marginal distributions of model parameters obtained from 1000 samples from the posterior mode *Q*_*I*_. Plots with red frames indicate strong and signifiant negative correlation (Pearsons’ −0.25 > *ρ* > 0.25, *p* ≪ 0.001).
(TIF)

S2 Fig Sensitivity analysis for posterior mode *Q*_*I*_. (TIF)

S3 Fig Local spread of chikungunya as predicted with *Q*_*C*_. (TIF)

S4 Fig Posterior modes *Q*_*I*_, *Q*_*C*_, *Q*_*S*_, and *Q*_*L*_. Pairwise marginal distributions of model parameters obtained from 1000 samples from the posterior modes *Q*_*I*_ (black), *Q*_*C*_ (red), *Q*_*S*_ (blue), and *Q*_*L*_ (green).
(TIF)
