# Supplementary material for: A large-scale stochastic spatiotemporal model for Aedes albopictus-borne chikungunya epidemiology
Source: PLoS One. 2017 Mar 31;12(3):e0174293. doi: 10.1371/journal.pone.0174293 (PMC5375158; doi:10.1371/journal.pone.0174293)
Supplement: S1 Text — Derivation of daily survival probability from gamma-distributed survival time. (PDF) [file pone.0174293.s001.pdf]

Supporting Text S1: Age-dependent survival

In models of age-independent survival, daily survival probability depends on environmental factors, such as temperature, but not on the age of vectors. With age-dependent survival, we assumed that the survival time of adult vectors is described by a gamma distribution [1]. In other words, we let the probability of surviving precisely x days be described by a gamma distribution.

According to this, daily survival probability of a vector of age d days can be calculated as complementary to the ratio of the probability of x being between day d and $d+1$ to the total probability of surviving at least d days,

$$p_d = 1 - \frac{\Pr(d < x \leq d+1)}{\Pr(d < x)},$$

where p_d represents daily survival probability in a stochastic context, or the daily expected fraction of survivors in a deterministic context.

The survival probability can be re-written as

$$p_d = 1 - \frac{F(d+1;\mu,\sigma) - F(d;\mu,\sigma)}{1 - F(d;\mu,\sigma)},$$

where $F(\cdot)$ is the cumulative distribution function of the gamma distribution with shape and scale parameters corresponding to a mean of μ days and a standard deviation of σ days.

Gamma distribution can be parameterised differently in different contexts. For instance, in GNU Scientific Library [2], it is implemented with a shape parameter k and a scale parameter θ, for which the first and second moments are $k\theta$ and $k\theta^2$, respectively. In this context, we chose to use mean, μ, and standard deviation, σ, as biologically meaningful parameters to describe the distribution, and calculated k and θ to match with the given μ and σ.

References

1. Wearing HJ, Rohani P, Keeling MJ (2005) Appropriate models for the management of infectious diseases. PLoS Med 2: e174.
2. Galassi M, et al. Gnu scientific library reference manual (3rd ed.). ISBN 0954612078. URL http://www.gnu.org/software/gsl.
