# Supplementary material for: A large-scale stochastic spatiotemporal model for Aedes albopictus-borne chikungunya epidemiology
Source: PLoS One. 2017 Mar 31;12(3):e0174293. doi: 10.1371/journal.pone.0174293 (PMC5375158; doi:10.1371/journal.pone.0174293)
Supplement: S2 Text — A brief analysis of the albopictus model posterior mode Θ4. (PDF) [file pone.0174293.s002.pdf]

# Supporting Text S2: Posterior mode Θ4 for *Aedes albopictus*

The posterior mode Θ4 was sampled using the same methodology, prior information, and observational data presented in Erguler *et al.* 2016 [1]. In essence, we used the `hoppMCMC` algorithm (v.0.5) of Python to perform parameter inference with prior information from the literature (see [1] for a comprehensive list) and observational data from the 7 provinces of Emilia-Romagna, namely Bologna, Modena, Parma, Reggio Emilia, Ferrara, Piacenza, and Ravenna [2].
In this version of the population dynamics model, we assumed a gamma-distributed life span for adult mosquitoes in contrast to fixed daily survival. In order to implement this, we extracted mean adult survival times, $\mu$, from the literature (Table 1) and assumed a fixed standard deviation of $0.375\mu$, which corresponds to the empirical standard deviation in the data.
Samples from the resulting posterior mode, Θ4, which also includes the average adult survival time, can be seen in Figure 1.

**Table 1. Reports of adult *Ae. albopictus* survival times from the literature.** Labels correspond to the respective data points in Figure 1 - adult survival times.

| Label | Reference | Label | Reference |
|---|---|---|---|
| 1 | [3] | 6 | [8] |
| 2 | [4] | 7 | [9] |
| 3 | [5] | 8 | [10] |
| 4 | [6] | 9 | [11] |
| 5 | [7] | 10 | [12] |

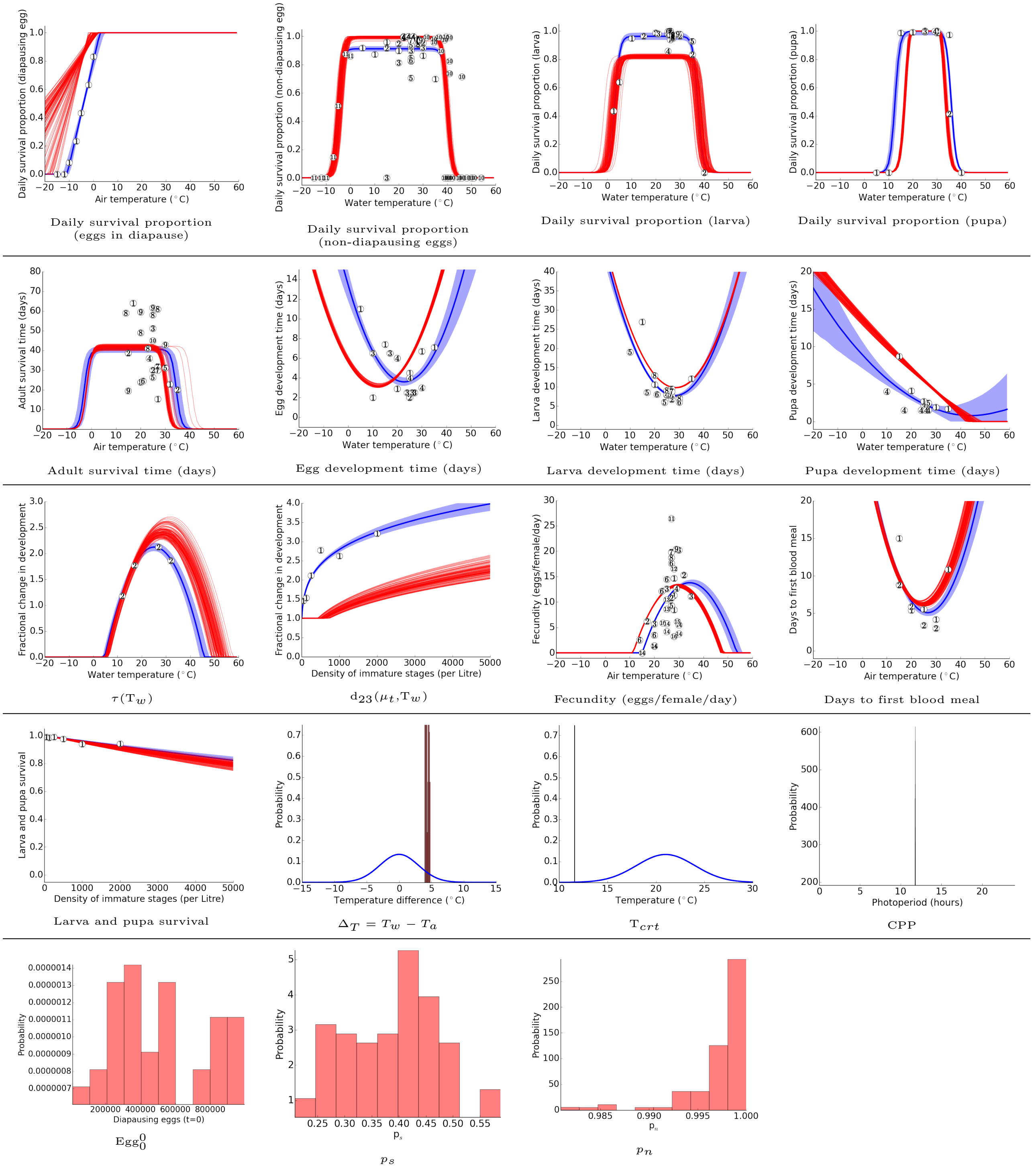


**Figure 1. Comparison of the prior and the posterior, Θ4, distribution of model parameters and the resulting functional forms.** Samples ($n = 100$) from the posterior distribution are plotted as red lines or histograms. Where applicable, the median, blue line, and the 95% range, blue shade, of the prior distribution are also plotted. The numbered dots represent data obtained from the literature (see Table 1 and S1 Table in [1]).

Further evaluation of the resulting posterior mode indicated that with regards to its biological implications it closely resembles the posterior mode Θ1 in Erguler *et al.* 2016 [1]. That is, Θ4 also suggests high cold resistance for diapausing eggs and the involvement of both temperature and photoperiod in diapause control. In contrast to Θ1, Θ4 emphasises the role of temperature in diapausing where the thresholds for photoperiod and temperature are $11.78 \pm 0.001$ hours and $11.59 \pm 0.003$ $°C$, respectively. The environmental dependence of diapause control can be summarised in Table 2.

**Table 2. Environmental dependency of diapause control as predicted with Θ4.** The numbers are percentages over all entry or exit events encountered during 2007-2012 in the 7 provinces of Emilia-Romagna.

| | Θ4 | | Θ1 | |
|---|---|---|---|---|
| | **Entry** | **Exit** | **Entry** | **Exit** |
| **Photoperiod** | 0 | 60.14 | 11.98 | 100 |
| **Temperature** | 100 | 23.19 | 75.77 | 0 |
| **Both** | 0 | 16.67 | 12.25 | 0 |

In Table 3, we list the correlation of simulated and observed egg counts over the surveillance region. In Table 4, we compare predicted habitat suitability over Europe to the presence reports from VBORNET [13]. The habitat suitability was calculated as the ratio of daily average adult female count to the minimum of this value calculated for the 7 provinces (see [1] for a detailed account of the habitat suitability index - HSI).

**Table 3. Agreement between observed and simulated egg counts.** Simulations were performed using the Θ4 posterior samples (n=100). Pearson correlation coefficients ($\rho$) are presented with p-values adjusted with Benjamini & Hochberg multiple test correction.

| | |
|---|---|
| **Bologna** | 0.645* |
| **Ferrara** | 0.630* |
| **Modena** | 0.753* |
| **Piacenza** | 0.669* |
| **Parma** | 0.789* |
| **Ravenna** | 0.560* |
| **Reggio Emilia** | 0.656* |
| **All data points** | 0.607* |

***** $p < 0.001$.

**Table 4. Validity of the habitat suitability analysis with Θ4 with respect to vector presence as reported by VBORNET.**

| **Trace** | **1/16** | **1/8** | **1/4** | **1/2** | **$\geq$ 1** |
|---|---|---|---|---|---|
| 1.1% | 0% | 1.1% | 0.8% | 10.5% | 86.5% |

In Figure 2, we compare the predicted and observed egg counts per ovitrap for the 7 provinces during 2008-2012. Finally, in Figure 3, we present the corresponding adult female counts per ovitrap as predicted by the population dynamics model with Θ4.

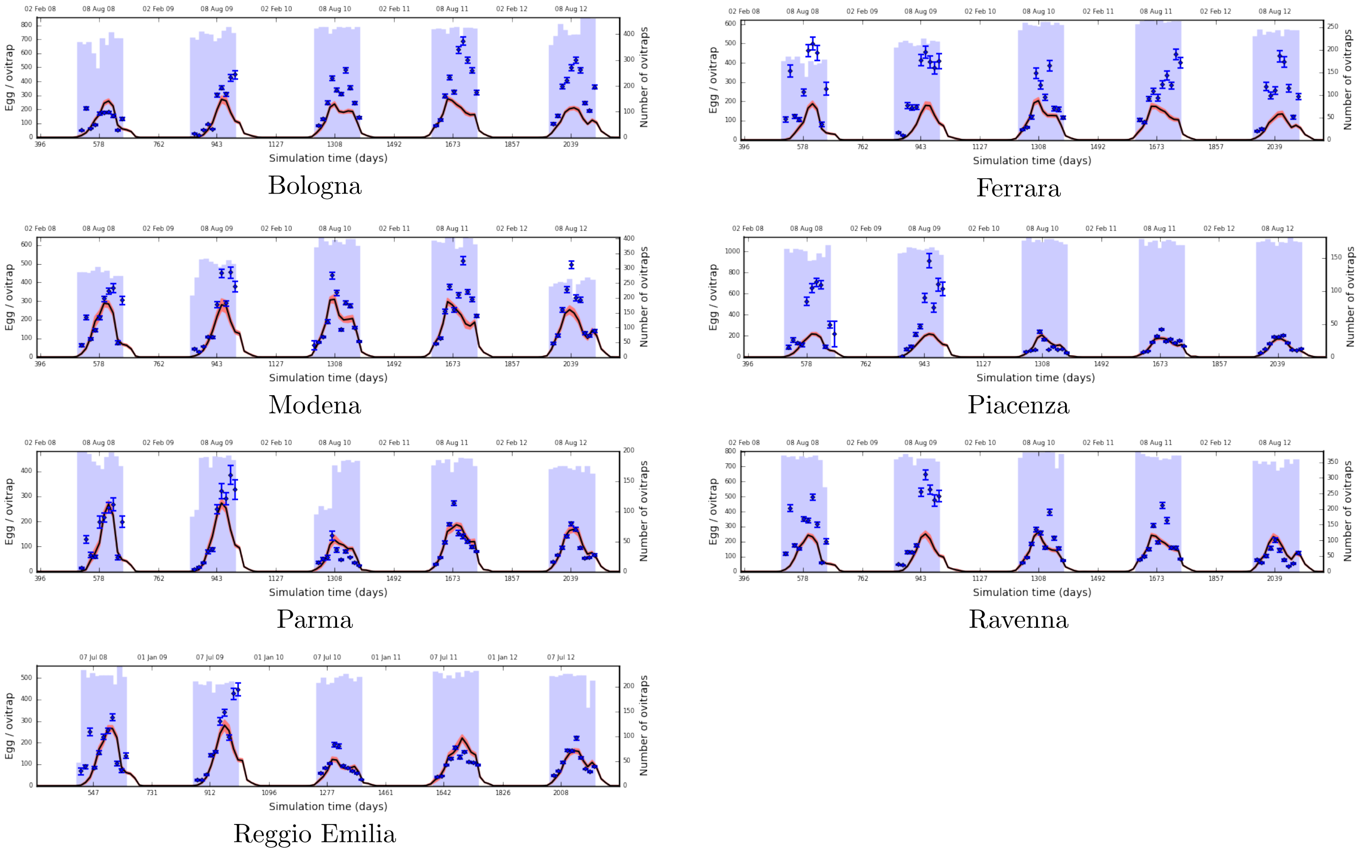


**Figure 2. Evaluating model performance over Emilia-Romagna with $\Theta 4$.** Blue diamonds represent average egg counts per ovitrap, and vertical error bars represent the standard error of the mean. They are positioned at the dates of data collection along the horizontal axis. Blue bars in the background indicate the number of ovitraps covering a period of two weeks prior to each collection. Solid black lines show model output using the parameters from $\Theta 4$. Red shades represent the 95% credible interval.

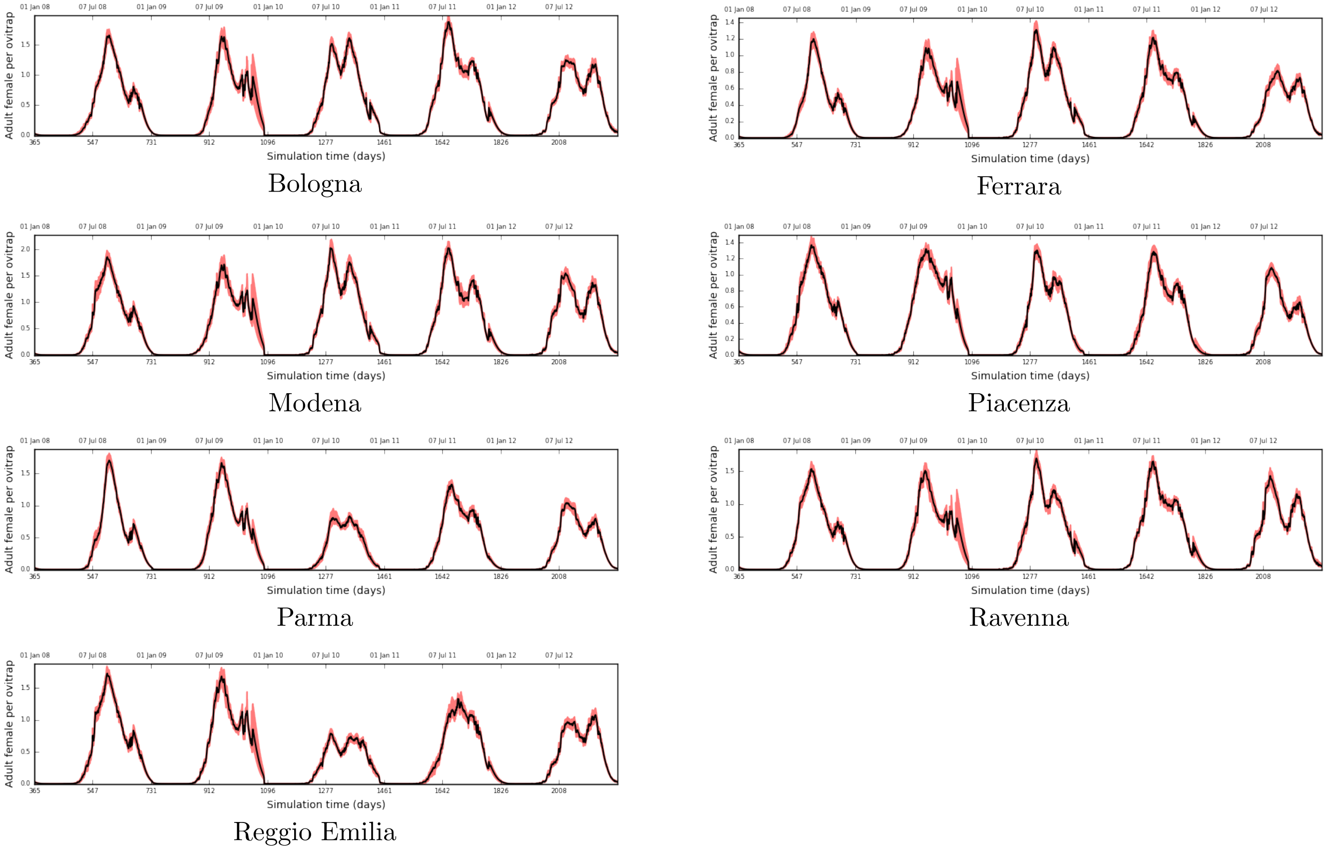


**Figure 3. Simulated number of adult females per ovitrap.** Simulations were performed with Θ4. Solid black lines indicate the mean while red shades delineate the 95% credible interval.

# References


1. Erguler K, Smith-Unna SE, Waldock J, Proestos Y, Christophides GK, et al. (2016) Large-scale modelling of the environmentally-driven population dynamics of temperate aedes albopictus (skuse). PLoS ONE 11: e0149282.
2. Carrieri M, Albieri A, Angelini P, Baldacchini F, Venturelli C, et al. (2011) Surveillance of the chikungunya vector aedes albopictus (skuse) in emilia-romagna (northern italy): organizational and technical aspects of a large scale monitoring system. J Vector Ecol 36: 108–16.
3. Briegel H, Timmermann SE (2001) Aedes albopictus (diptera: Culicidae): Physiological aspects of development and reproduction. Journal of Medical Entomology 38: 566-571.
4. Delatte H, Gimonneau G, Triboire A, Fontenille D (2009) Influence of temperature on immature development, survival, longevity, fecundity, and gonotrophic cycles of aedes albopictus, vector of chikungunya and dengue in the indian ocean. Journal of Medical Entomology 46: 33-41.
5. Braks MAH, Juliano SA, Lounibos LP (2006) Superior reproductive success on human blood without sugar is not limited to highly anthropophilic mosquito species. Medical and veterinary entomology 20: 53–59.
6. Löwenberg Neto P, Navarro-Silva MA (2004) Development, longevity, gonotrophic cycle and oviposition of aedes albopictus skuse (diptera: Culicidae) under cyclic temperatures. Neotropical Entomology 33: 29-33.
7. Muturi EJ, Lampman R, Costanzo K, Alto BW (2011) Effect of temperature and insecticide stress on life-history traits of culex restuans and aedes albopictus (diptera: Culicidae). Journal of Medical Entomology 48: 243-250.
8. O'Donnell D, Armbruster P (2010) Inbreeding depression affects life-history traits but not infection by plasmodium gallinaceum in the asian tiger mosquito, aedes albopictus. Infection, Genetics and Evolution 10: 669–677.

9. Tsuda Y, Takagi M, Suzuki A, Wada Y (1994) A comparative study on life table characteristics of two strains of aedes albopictus from japan and thailand. Tropical Medicine 36.
10. Udaka M (1959) Some ecological notes on aedes albopictus skuse in shikoku, japan. Japanese journal of entomology 27: 202-208.
11. Calado DC, Navarro-Silva MA (2002) Influência da temperatura sobre a longevidade, fecundidade e atividade hematofágica de aedes (stegomyia) albopictus skuse, 1894 (diptera, culicidae) sob condições de laboratório. Rev Bras Entomol 46: 93–98.
12. Hien DS (1975) Biology of aedes aegypti (l. 1762) and aedes albopictus (skuse, 1895) (diptera, culicidae). II. effect of certain environmental conditions on the hatching of larvae. Acta Parasitol Pol 23: 537-552.
13. (October 2015). Network of medical entomologists and public health experts. ECDC/VBORNET. URL http://www.ecdc.europa.eu/en/healthtopics/vectors/vector-maps/Pages/VBORNET_maps.aspx.
