# Supplementary figures and images for: A large-scale stochastic spatiotemporal model for Aedes albopictus-borne chikungunya epidemiology
Source: PLoS One. 2017 Mar 31;12(3):e0174293. doi: 10.1371/journal.pone.0174293 (PMC5375158; doi:10.1371/journal.pone.0174293)

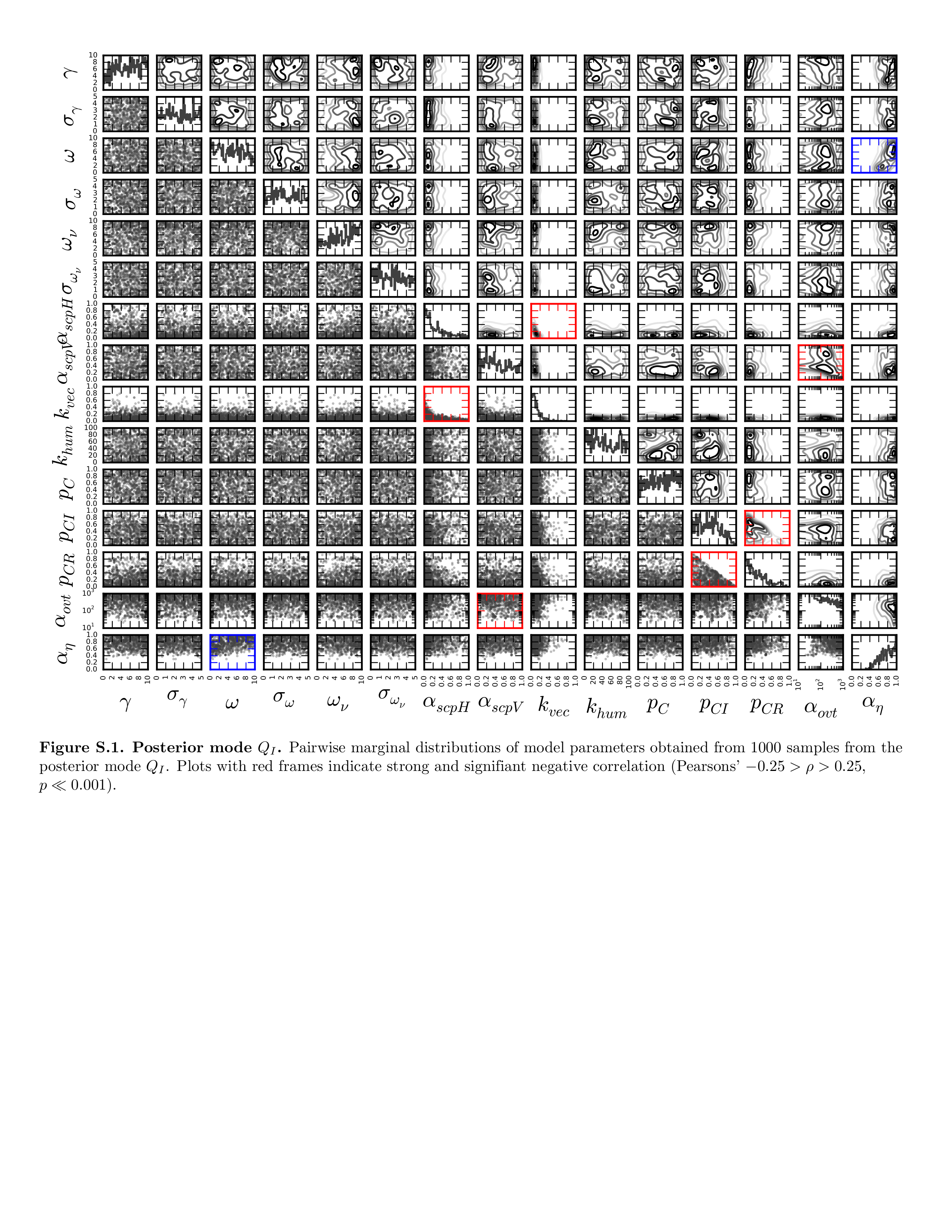

Supplement: S1 Fig — Pairwise marginal distributions of model parameters obtained from 1000 samples from the posterior mode QI. Plots with red frames indicate strong and signifiant negative correlation (Pearsons’ −0.25 > ρ > 0.25, p ≪ 0.001). (TIF) [file pone.0174293.s004.tif]

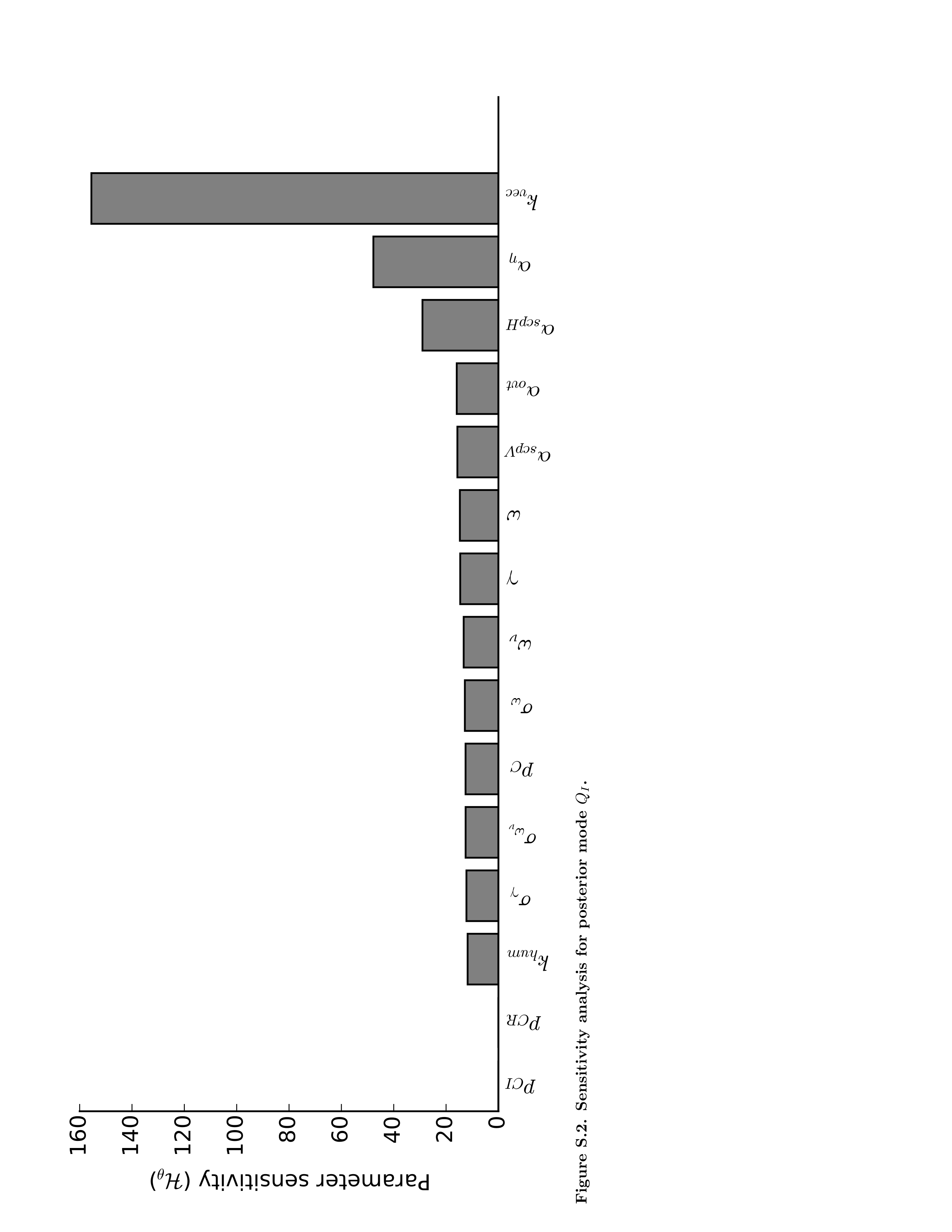

Supplement: S2 Fig — (TIF) [file pone.0174293.s005.tif]

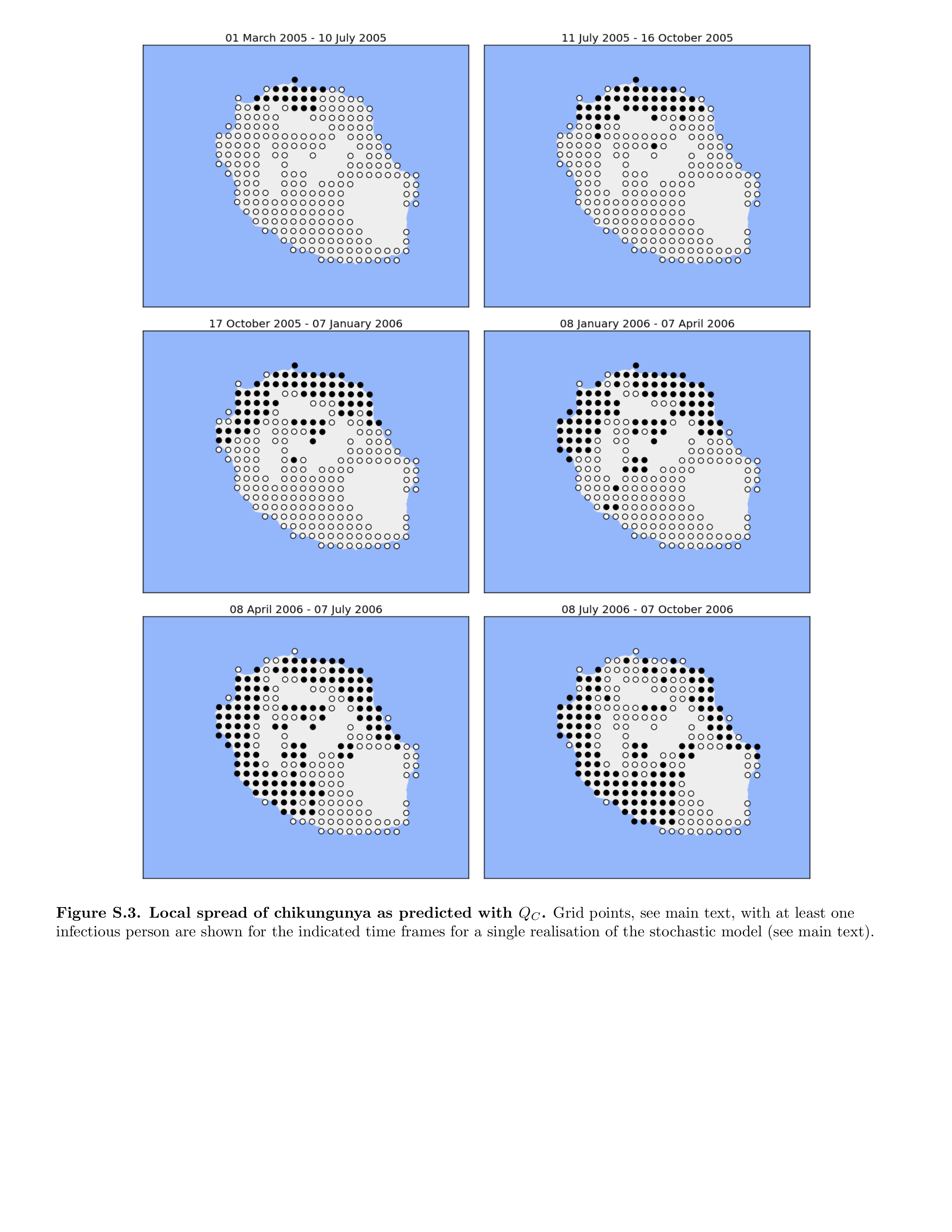

Supplement: S3 Fig — (TIF) [file pone.0174293.s006.tif]

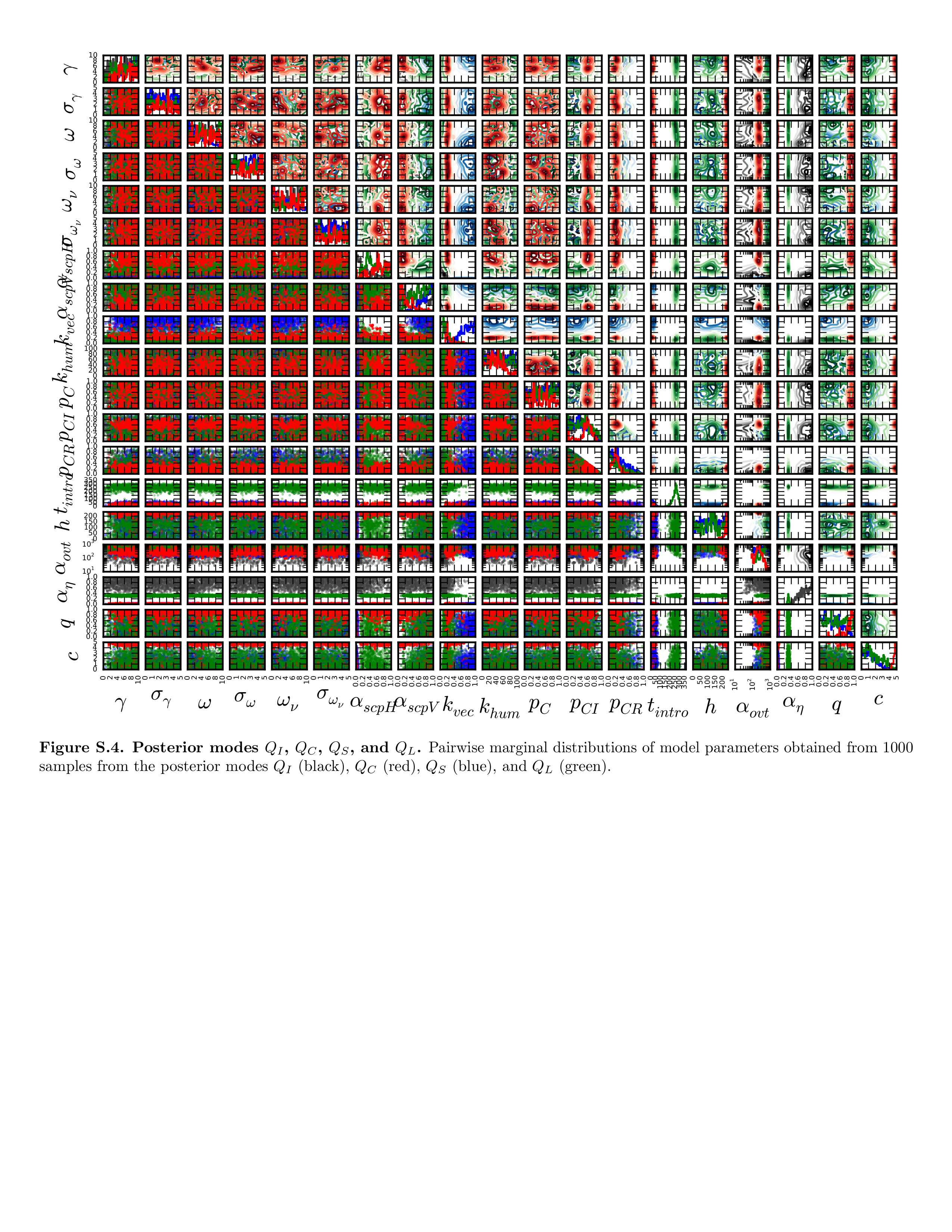

Supplement: S4 Fig — Pairwise marginal distributions of model parameters obtained from 1000 samples from the posterior modes QI (black), QC (red), QS (blue), and QL (green). (TIF) [file pone.0174293.s007.tif]
